# Vascular Plant One-Zinc-Finger (VOZ) Transcription Factors Are Positive Regulators of Salt Tolerance in Arabidopsis

**DOI:** 10.3390/ijms19123731

**Published:** 2018-11-23

**Authors:** Kasavajhala V. S. K. Prasad, Denghui Xing, Anireddy S. N. Reddy

**Affiliations:** 1 Department of Biology and Cell and Molecular Biology Program, Colorado State University, Fort Collins, CO 80523, USA; kpsatya@mail.colostate.edu (K.V.S.K.P.); david.xing@mso.umt.edu (D.X.); 2 Genomics Core Lab, Division of Biological Sciences, University of Montana, Missoula, MT 59812, USA

**Keywords:** *Arabidopsis thaliana*, VOZ, transcription factor, salt stress, transcriptional activator

## Abstract

Soil salinity, a significant problem in agriculture, severely limits the productivity of crop plants. Plants respond to and cope with salt stress by reprogramming gene expression via multiple signaling pathways that converge on transcription factors. To develop strategies to generate salt-tolerant crops, it is necessary to identify transcription factors that modulate salt stress responses in plants. In this study, we investigated the role of VOZ (VASCULAR PLANT ONE-ZINC FINGER PROTEIN) transcription factors (VOZs) in salt stress response. Transcriptome analysis in WT (wild-type), *voz1-1*, *voz2-1* double mutant and a *VOZ2* complemented line revealed that many stress-responsive genes are regulated by VOZs. Enrichment analysis for gene ontology terms in misregulated genes in *voz* double mutant confirmed previously identified roles of VOZs and suggested a new role for them in salt stress. To confirm VOZs role in salt stress, we analyzed seed germination and seedling growth of WT, *voz1*, *voz2-1*, *voz2-2* single mutants, *voz1-1* *voz2-1* double mutant and a complemented line under different concentrations of NaCl. Only the double mutant exhibited hypersensitivity to salt stress as compared to WT, single mutants, and a complemented line. Expression analysis showed that hypersensitivity of the double mutant was accompanied by reduced expression of salt-inducible genes. These results suggest that VOZ transcription factors act as positive regulators of several salt-responsive genes and that the two VOZs are functionally redundant in salt stress.

## 1. Introduction 

Throughout their life span, plants are constantly subjected to diverse abiotic and biotic stresses, which severely inhibit plant growth and development, and cause huge losses in crop yields [1,2]. As sessile organisms, plants respond to these stresses rapidly by altering their gene expression patterns, which ultimately change biochemical and physiological processes that enable them to survive under stress conditions [2,3,4]. Plant transcription factors (TFs) play a key role in reprogramming gene expression in response to stresses [3,4,5,6]. Many of these transcription factors act by regulating the expression of down-stream genes that are important for stress tolerance [2,3,4,7]. VOZ (VASCULAR PLANT ONE-ZINC FINGER PROTEIN) is a plant-specific TF family with two members, *VOZ1* and *VOZ2*. Previous studies have shown that *VOZ1* is specifically expressed in the phloem tissue while *VOZ2* is highly expressed in the roots [8]. Yasui et al. [9] have shown their localization in vascular bundles and predominant subcellular presence in the cytoplasm, while they function in the nucleus. VOZ TFs (for brevity we refers to them as VOZs) were identified as proteins that bind to a *cis*-element *GCGTNx7ACGC* in the promoter of *AVP1* (V-PPAse) [8]. VOZs have two conserved domains (viz., A and B) and share about 53% similarity. VOZ2 regulates the expression of the target genes by binding to *cis-*elements via Domain B as a dimer. The B domain has a zinc finger motif and a basic region [8].

VOZs were also classified into NAC (for NAM (no apical meristem)) subgroup VIII-2 as they share homology with NAC proteins in the C-terminal basic region [10]. VOZs regulate flowering through their interaction with *PHYB* and promote the expression of *FLC* and *FT* [9,11]. More recent genetic, biochemical and cell biological studies have shown that VOZs interact with and modulate the function of CONSTANS (CO) in promoting flowering [12]. VOZs also play a key role in plant responses to abiotic (cold, drought and heat) and biotic (pathogens) stresses. VOZs function as a positive regulator of plant responses against bacterial and fungal pathogens, and as a negative regulator of two abiotic stresses-old and drought [13,14]. The expression levels of both *VOZ1* and *VOZ2* were also altered in response to biotic and abiotic stresses in an opposite manner [13]. Overexpression of the VOZ2 conferred biotic stress tolerance, however it showed sensitivity to freezing and drought stress in Arabidopsis [13]. Recent reports indicate that VOZ1 and VOZ2 act as transcriptional repressors for *DREB2C* and *DREB2A* respectively, which mediate heat stress response in plants [15,16]. Despite a few reports describing the role of VOZs in some abiotic stresses [17,18,19,20], the full scope of VOZs’ function in other stresses and their potential target genes are not well understood. In the current study, analysis transcriptomes from WT, *voz1-1 voz2-1* double mutant and a complemented line suggested a new role for VOZs in salt stress. Analysis of seed germination and seedling growth of WT, *voz1*, *voz2-1*, *voz2-2* single mutants, *voz1-1 voz2-1* double mutant and a complemented line under different concentrations of NaCl revealed that only the double mutant is hypersensitive to salt stress. Through analysis of the upstream regions of genes regulated by VOZs for canonical and non-canonical binding sites, we have identified potential new targets of VOZ transcription factors. Furthermore, expression of salt-induced genes is impaired in the VOZ double mutant. Collectively, these results suggest that VOZs act as positive regulators of salt stress response and that the two VOZs are functionally redundant in salt stress.

## 2. Results

**Figure 1:**
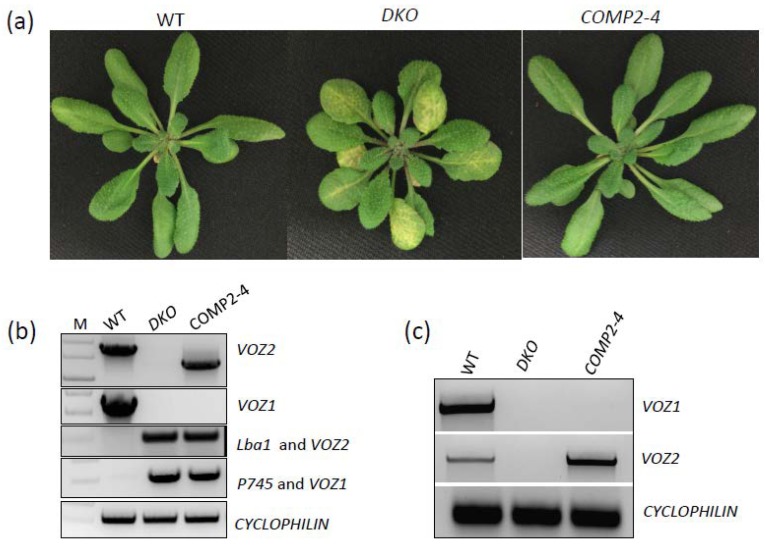
Validation of genotypes used for RNA-Seq. (**a**) Top panel: Phenotype of 30-day-old plants of wild-type (WT), double knockout (*DKO)* mutant (*voz1-1 voz2-1*) and *DKO* complemented line (COMP2-4) grown at 21 °C under day neutral conditions at 60% humidity. (**b**) Genomic PCR of three genotypes used for RNA-Seq. Top panel (PCR with *VOZ2*-specific primers); second panel (PCR with *VOZ1*-specific primers); third panel (PCR with T-DNA specific Lba1 and *VOZ2*-specific reverse primer); fourth panel (PCR with Tn insertion specific primer P745 and VOZ1-specific forward primer); bottom panel (PCR with *CYCLOPHILIN*-specific primers). In all cases expected size PCR product was obtained. (**c**) Analysis of expression of *VOZ1* (top panel), *VOZ2* (middle panel) and *CYCLOPHILIN* (bottom panel) using sqRT-PCR in 30-day-old seedlings of WT, *DKO* mutant (*voz1-1 voz2-1*) and *DKO* complemented line (COMP2-4).

Arabidopsis VOZ TF family contains two members—*VOZ1* and *VOZ2*. Both these TFs were reported to be involved in flowering and response to abiotic (cold, drought and heat) and biotic stresses [9,11,12,13,14,15,16]. Here we performed RNA-Seq analysis of gene expression with RNA from wild type (WT), a *voz* double knockout (DKO—*voz1-1 voz2-1*) mutant and a complemented line (COMP2-4). Double mutant (DKO) lines exhibited suppressed growth and leaf vein clearing in older leaves in comparison to WT and COMP2-4 line (Figure 1a). Expression of *VOZ2* in *voz1-1 voz2-1* (COMP2-4) rescued the DKO phenotype (Figure 1a). Hence, we have chosen 30-day-old plants to identify new potential targets of the VOZs by comparing the transcriptomes of WT, DKO and complemented line (COMP2-4). Prior to RNA-Seq and phenotypic analysis, the genotypes of all the lines were verified by genomic PCR (Figure 1b and Figure S1a–c) with gene-specific and T-DNA or transposon-specific primers. RT-PCR analysis with *VOZ1* and *VOZ2* specific primers confirmed the absence of transcripts in *DKO* line (Figure 1c).

### 2.1. Loss of Function of VOZs Resulted in Misregulation of Genes

**Figure 2:**
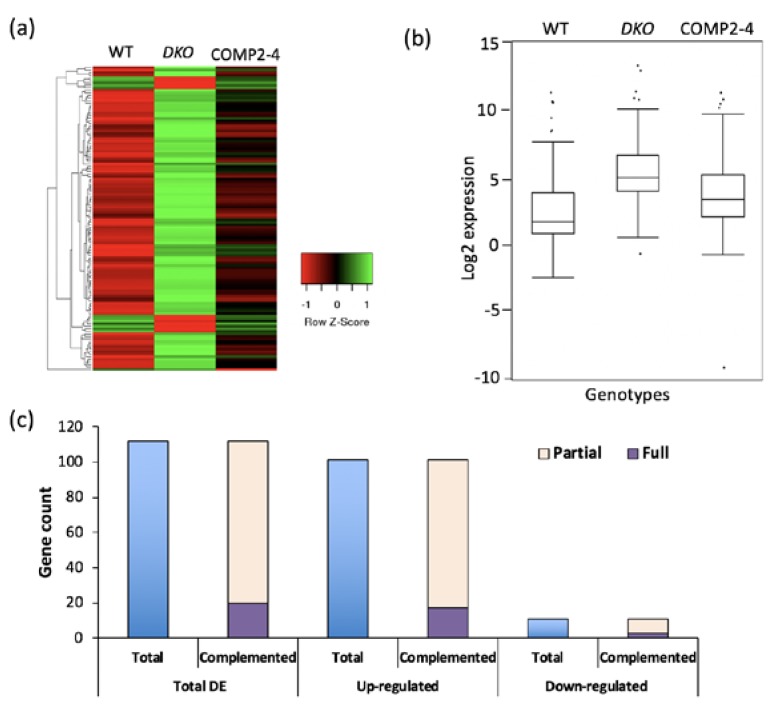
Analysis of differentially expressed genes. (**a**) Heatmap representation of differentially expressed genes in *WT*, *DKO* and *COMP2-4* (COMP) plants. Expression values were used to generate the heatmap using the Heatmapper. Columns represent samples and rows represent genes. Color scale indicates the gene expression level. Green indicates high expression and red Indicates low expression. (**b**) Box-and-whisker plots showing expression of differentially expressed (DE) genes in different genotypes. (**c**) Gene counts of total, up and down-regulated DE genes that are either fully or partially complemented in *COMP2-4* line.

**Figure 3:**
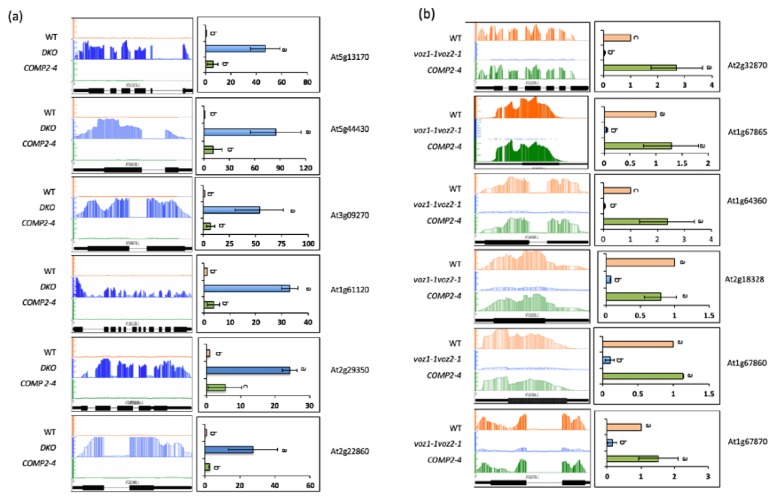
Validation of up- and down-regulated genes in *DKO*. (**a**) RT-qPCR validation of randomly selected up-regulated genes. (**b**) RT-qPCR of randomly selected down-regulated genes. Left panels in (**a**,**b**) show relative sequence read abundance (Integrated Genome Browser view) as histograms in WT, *DKO (voz1-1 voz2-1)* and COMP2-4 lines. The Y-axis indicates read depth with the same scale for all three lines. The gene structure is shown below the read depth profile. The lines represent introns and the boxes represent exons. The thinner boxes represent 5′ and 3′ UTRs. Right panels in (**a**,**b**) show fold change in expression level relative to WT. WT values were considered as 1. Student’s *t*-test was performed and significant differences (*p* < 0.05) among samples are labeled with different letters. The error bars represent SD. The genes that were randomly picked include At1g61120 (terpene synthase 4), At1g64360 (enescence-associated and QQS-related), At1g67860 (hypothetical protein), At1g67865 (hypothetical protein), At1g67870 (hypothetical protein), At2g18328 (RAD-like4), At2g22860 (phytosulfokine 2 Precursor), At2g29350 (senescence-associated gene 13), At3g09270 (glutathione S-transferase TAU8), At2g32870 (TRAF-like protein), At5g13170 (senescence-associated gene29), and At5g44430 (plant defensing 1.2C).

For RNA-Seq, two biological replicates of WT, *DKO* and COMP2-4 were used. About 37 to 75 million short reads per sample were obtained (Table S1). The reads were mapped to the Arabidopsis genome (TAIR 10) and ~90% of these were uniquely mapped (Table S1). The expression levels of individual transcripts were determined by the number of reads per kilobase per million (RPKM). The expression patterns of the genes were well correlated among the replicates. However, the expression patterns were poorly correlated between WT and *DKO*, as indicated by an R^2^ value of 0.77, suggesting a significant effect of VOZs on gene expression (Figure S2). The Cufflinks package was used to identify differentially expressed (DE) genes by comparing the transcriptome of WT with *DKO*. In the *DKO*, 112 genes were misregulated (significance adj. *p* < 0.05 and fold change >log2) as compared to WT (Additional File 1 sheet1). Further, expression levels of the majority of these were either partially or fully restored in the *VOZ2* complemented line (COMP2-4) (Figure 2a–c; Additional File 1 sheet2), suggesting that DE genes are either direct or indirect targets of VOZs and loss of these TFs caused significant effects on expression of many genes. The majority of the DE genes (101) were up-regulated, while only 11 were down-regulated in the *DKO* mutant. About 83% of up-regulated genes are partially complemented while ~27% of them are fully complemented by overexpression of *VOZ2*. In the case of down-regulated DE genes, ~27% of genes were fully complemented and 72% exhibited partial complementation (Figure 2). The misregulation of a number of DE genes was verified using RT-qPCR (Figure 3a,b), corroborating the RNA-Seq data.

### 2.2. VOZs Regulate Expression of Several Transcription Factors

**Figure 4:**
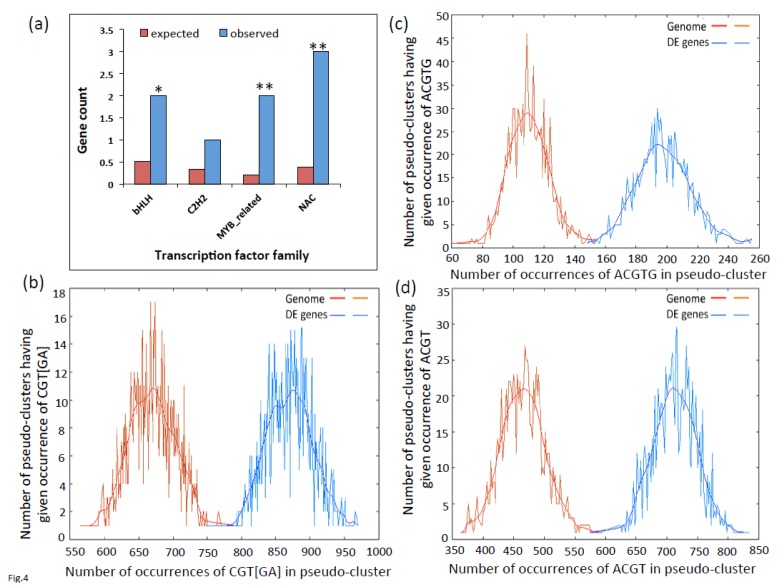
Enrichment of transcription factor families and VOZ binding sites in the promoters of DE genes. (**a**) DE genes are enriched for specific TF families. Observed: Number of genes associated with particular TF family in DE genes. Expected: Number of genes expected in each individual TF family in the genome. Asterisks on the bar represent significant overrepresentation of TFs with a (* *p* ≤ 0.05) and (** *p* ≤ 0.0001), respectively. (**b**–**d**) POBO analysis of NAC consensus sequence (*CGT[GA]*), G-box core sequence (*ACGTG*) and LS-7 *cis*-element (*ACGT*), respectively, in the −1000 bp upstream of TSS. One thousand pseudoclusters were generated from top 112 DE genes and genome background. The jagged lines show the motif frequencies from which the best-fit curve is derived. *CGT*[*GA*], *ACGTG* and *ACGT* elements are significantly overrepresented (two-tailed *p* < 0.0001) in the upstream sequences of DE genes.

It is possible that the effect of VOZs on the expression of its DE genes is mediated via its regulation of other TFs, hence we analyzed the DE genes for enrichment of TFs. Arabidopsis has over 1700 genes encoding TFs that are grouped into 58 families. Among DE genes, we observed eight TFs belonging to four families (Figure 4a, Additional File 2 and Figure S3). Of these families, bHLH (Basic-Helix-Loop-Helix) (*p* ≤ 0.03), MYB (v-myb avian myeloblastosis viral oncogene homolog)-related (*p* ≤ 0.00009) and NAC (*p* ≤ 0.00002) are highly enriched (Figure 4a). The number of TFs in each family and the direction of their expression in the *DKO* are presented in Figure S3 and Additional File 2. Interestingly, members of bHLH, NAC and C_2_H_2_ TF families are up-regulated whereas the members of MYB-related families are down-regulated in *DKO.* Significantly, five out of eight members of TF families showed expression levels similar to that of WT in the complemented line, indicating that VOZs regulates the expression of these transcription factors.

### 2.3. Promoters of Differentially Expressed Genes are Enriched for G-Box, NAC and LS-7 Elements

**Figure 5:**
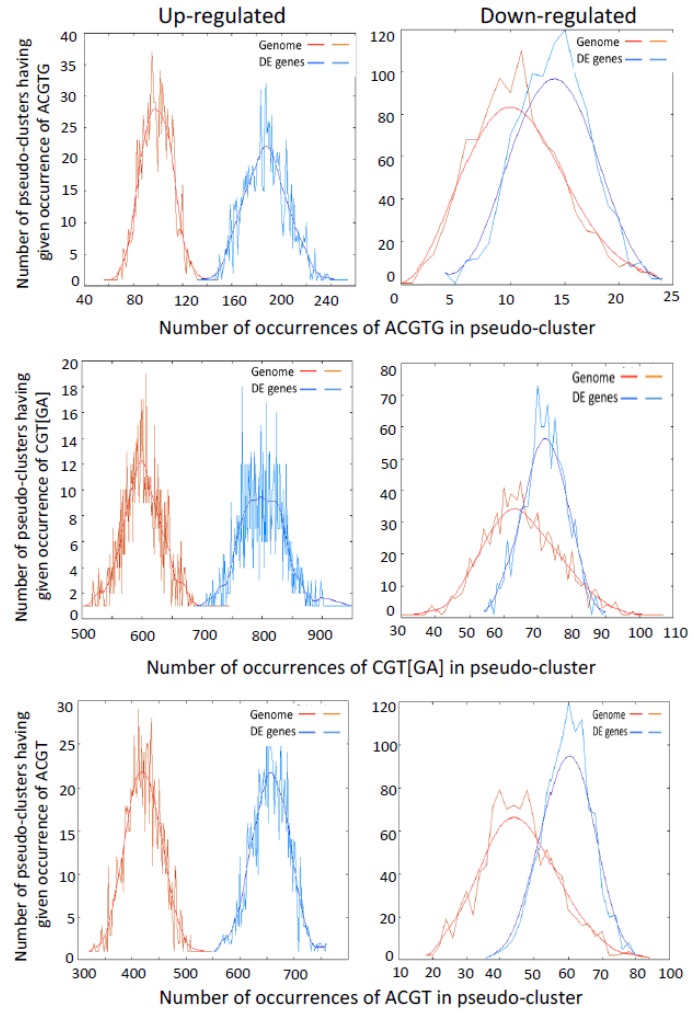
VOZ-binding sites in the promoters of up- and down-regulated DE genes. POBO analysis of VOZs binding motif, G-box core sequence (ACGTG) (top panels), NAC consensus sequence (CGT[GA]) (middle panels), and LS-7 *cis*-element (ACGT) (bottom panels) in the −1000 bp upstream of TSS. A total of 1000 pseudoclusters were generated from 101 up-regulated (left panels) and 11 down-regulated genes (right panels) and genome background. The jagged lines show the motif frequencies from which best-fitted curve is derived. VOZs binding sites are significantly (two-tailed *p* < 0.0001) over-represented in the upstream sequences of both up- and down-regulated genes.

As VOZs share significant sequence similarity with NAC subgroup VIII-2 TFs (particularly in the C-terminal region) and also reported to bind to the palindromic NAC binding sequence (palNAC-BS), we analyzed the promoters of DE genes (−1000 bp upstream of TSS) for the enrichment for G-box core sequence (*ACGTG*), NAC-consensus sequence (*CGT[GA]*) and TGA TFs recognition LS-7 element (*ACGT*), which are thought to bind VOZs, using POBO analysis. This analysis revealed a significant enrichment of *cis*-elements (*p* < 0.0001) of *ACGTG*, *CGT[GA]* and *ACGT* in the promoter regions of DE genes (Figure 4b–d). Ninety percent of the DE genes contain *CGT[GA]* (1 to 12), 58% have *ACGTG* (1 to 8) and 84% have *ACGT* (1 to 12) binding sites in their upstream (−500 bp) region (Table S2). Furthermore, POBO analysis of up- and down-regulated DE genes separately also exhibited significant (*p* ≤ 0.0001) enrichment of these binding sites (Figure 5). These results indicate VOZs might regulate the expression of some of these DE genes directly through these elements.

### 2.4. GO Term Enrichment Analysis for Biological Processes in Differentially Expressed Genes

**Figure 6:**
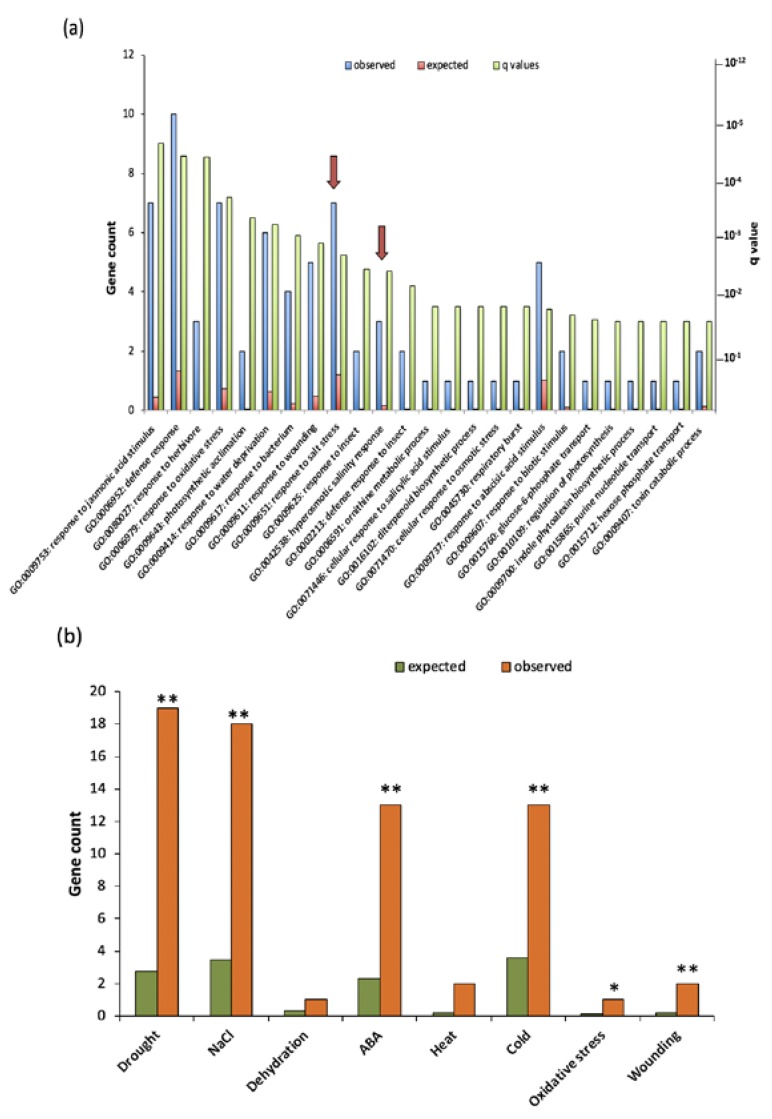
Gene ontology (GO) enrichment analysis of DE genes. (**a**) GO term enrichment analysis for biological processes of up-regulated genes. For each GO term, the expected and observed gene numbers along with the statistical significance (*p*-value) for the enrichment is presented. Observed: Number of DE genes associated with a GO term for biological processes. Expected: Number of genes expected for each GO term in the genome. “Response to salt stress” and “Hyperosmotic salinity response” GO terms are indicated with an arrow. (**b**) A significant number of DE genes are associated with abiotic stress response in comparison with genome background with a ** *p* ≤ 0.0001 and * *p* ≤ 0.05.

Previously, VOZ proteins have been shown to play an important role in flowering, plant immunity, cold, heat, and drought stresses [9,11,12,13,14,15,16,17]. We performed gene ontology (GO) analysis not only to verify if DE genes function in previously reported processes but also to gain insight into other functional roles of VOZs. A singular GO term enrichment analysis for biological processes was performed with GeneCodis separately for up-regulated and down-regulated DE genes. No significant enrichment of any GO term for down-regulated DE genes was found. However, a total of 38 GO terms for biological processes were enriched in up-regulated DE genes (Figure 6a and Additional File 3). Consistent with previously reported functions of VOZs, GO terms associated with the processes involving plant response to pathogen/pests and water deprivation, osmotic stress and oxidative stress were enriched. GO terms that are of special interest are “response to salt stress” and “hyperosmotic salinity response” for the following reasons: (i) both are among the top 10 most enriched GO terms; (ii) these two GO terms together have 10 genes (second most of all GO categories); (iii) role of VOZ proteins in salt stress is not known; and (iv) expression of the majority of these genes is altered in opposite direction in the complemented line (Table S3).

### 2.5. VOZs Regulate Expression of Many Abiotic Stress-Responsive Genes

Since G-box, NAC and TGA *cis*-elements occurred significantly in the promoter region of DE genes (Figure 4 and Figure 5), we performed enrichment analysis to determine the number of DE genes associated with different stresses. For this analysis, we compared the DE gene list with all the listed abiotic stress genes at http://caps.ncbs.res.in/stifdb/browse.html#se. This analysis also indicated a substantial enrichment (*p* ≤ 0.001) of different abiotic stress-responsive genes with a significant number of genes associated with drought and cold stress, which is consistent with the reported functions of VOZs (Figure 6b and Additional File 4) of Nakai et al. [14]. However, VOZ’s function in salt stress is not known. Enrichment analysis indicated that about 16% of DE genes (18 genes) in *DKO* are associated with salt stress (Additional File 4). Furthermore, in the COMP2-4 line, the expression of salt stress-responsive genes found in both these analyses was partially or fully restored to WT levels (Figure S5 and Table S3a,b).

### 2.6. VOZs Regulate Salt Stress Tolerance 

#### 2.6.1. VOZ Double Mutant Exhibits Hypersensitivity to Salt Stress

**Figure 7:**
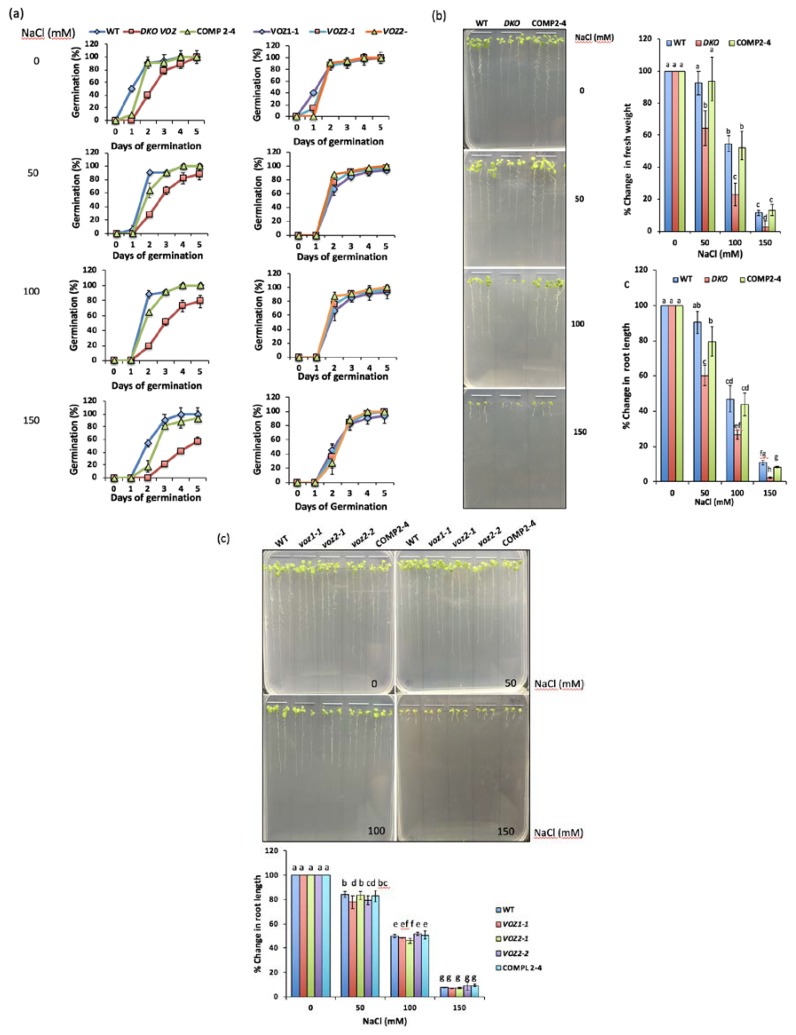
Germination and seedling growth of WT, mutants and complemented line in the presence of NaCl. (**a**) *VOZ* Double mutant (DKO) exhibits delayed germination under salt stress. The time course of seed germination of WT, DKO, COMP2-4 (left panels), *voz1-1*, *voz2-1* and *voz2-2* (right panels) in the presence of 0, 50, 100 and 150 mM NaCl. Each value shown here is mean of three biological replicates with *n* = 10. The error bars represent SD. (**b**) *VOZ* Double mutant (DKO) is hypersensitive to salt stress. Left panel: Growth of seedlings of WT, DKO and COMP2-4 on MS (Murashige and Skoog medium) plates containing different concentrations of NaCl. Seeds were plated on 1/2 strength MS medium supplemented with 0, 50, 100 and 150 mM of NaCl and were allowed to germinate and grow for two weeks. The photographs were taken after two weeks. Right panel, top: Seedling fresh weight. Right panel, bottom: Seedling root length at different concentrations of NaCl was measured for all genotypes and plotted as % relative to growth on normal (0 mM) MS medium. Three biological replicates were used. Eight to ten seedlings for each genotype per treatment for each biological replicate were included. Student’s *t*-test was performed and significant differences (*p* ≤ 0.05) among samples are labeled with different letters. The error bars represent SD. (**c**) Single mutants of VOZs are not hypersensitive to salt stress. Top: Growth of seedlings of WT, COMP2-4, *voz1-1*, *voz2-1* and *voz2-2* on MS plates containing different concentrations of salt. Seeds were plated on half-strength MS medium supplemented with 0, 50, 100 or 150 mM of NaCl and were allowed to germinate and grow for two weeks. The photographs were taken after two weeks. Bottom: Seedling root length at different concentrations of NaCl was measured for all genotypes and plotted as % relative to growth on normal (0 mM) MS medium. Three biological replicates were used. For each genotype, eight to ten seedlings per treatment and for each biological replicate were used. Student’s *t*-test was performed and significant differences (*p* ≤ 0.05) among samples are labeled with different letters. The error bars represent SD.

Previous studies have shown that VOZs play an important role in drought, cold and heat [13,15,16], but their regulatory role in salt stress is not known. Since salt stress-responsive genes are enriched in DE genes, we investigated the role of VOZs in salt stress tolerance. Wild type, double mutant (DKO), COMP 2-4 and single mutants of VOZs (*voz1-1*, *voz2-1*, *voz2-2)* were tested for salt tolerance. Seed germination rate, seedling growth and root length were scored by growing them on different concentrations (0, 100 or 150 mM) of NaCl. In general, seed germination rate was significantly affected under salt stress in a NaCl concentration-dependent manner in all genotypes. However, irrespective of NaCl concentration, seeds of DKO genotype exhibited delayed germination in comparison to WT, COMP2-4 and single *voz* mutants (Figure 7a). Further, seedling growth (fresh weight) was also significantly affected in a NaCl concentration-dependent manner in all the genotypes. Similar to the rate of seed germination, the growth of DKO seedlings was severely suppressed when compared to that of WT, COMP2-4 and single mutants (Figure 7b,c). A suppression of the primary root length in a NaCl concentration-dependent manner was also observed (Figure 7b). Particularly at 100 and 150 mM NaCl, a difference in root growth was evident among the genotypes (Figure 7b—left bottom panel and 7c). A significant suppression in the primary root growth was observed in DKO lines as compared to WT, COMP2-4 and single mutant lines, indicating the increased sensitivity of DKO mutant to salt stress (Figure 7b,c). Even at 150 mM NaCl, WT, COMP2-4 and single mutants were found to be relatively more tolerant to salt stress, indicating DKO seedlings exhibit hypersensitivity to salt stress.

#### 2.6.2. VOZs Activate the Expression of Salt-Responsive Genes

**Figure 8:**
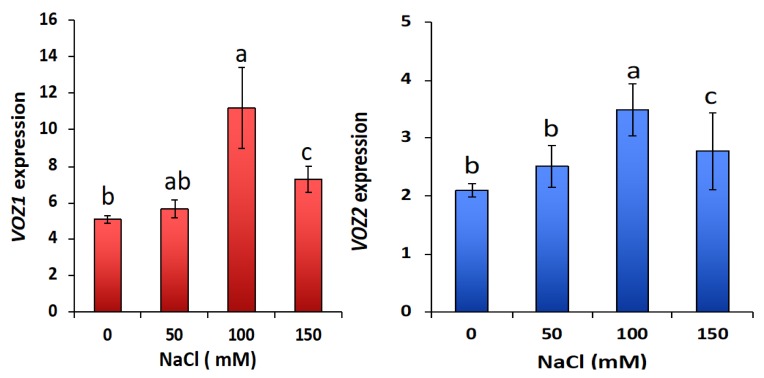
Expression of VOZs in response to salt stress. Expression of *VOZ1* (top panel) and *VOZ2* (bottom panel) in 10-day-old seedlings of WT seedlings grown on 1/2 MS medium supplemented with 0, 50, 100 or 150 mM NaCl was determined by RT-qPCR. The expression of *VOZs* was normalized with *ACTIN2*.

**Figure 9:**
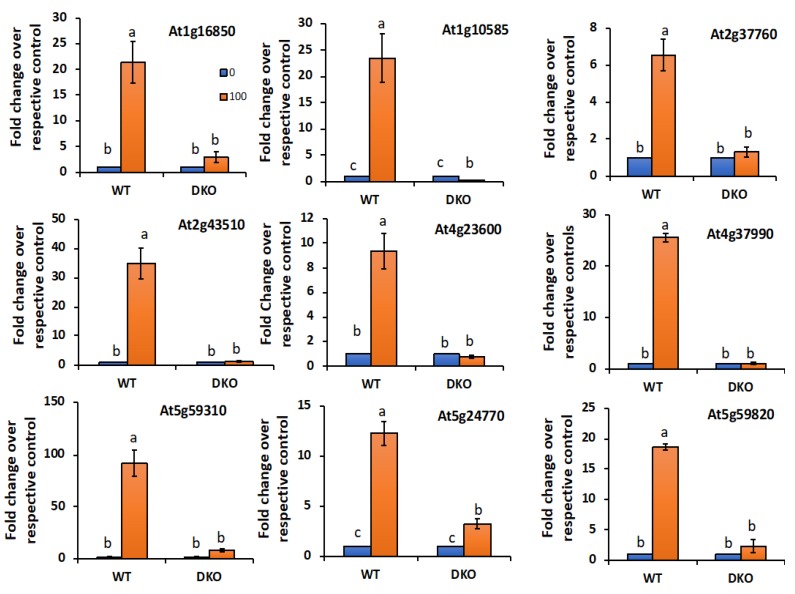
VOZs positively regulate the expression of salt-responsive genes. Expression of salt-responsive genes in 10-day-old seedlings of WT and DKO lines on 1/2 MS medium supplemented with 0 or 100 mM NaCl was determined by RT-qPCR. The expression level of salt-responsive genes was normalized with *ACTIN2*. Fold change in expression level relative to their respective controls (0 mM) is presented. 0 mM values were considered as 1. Three biological replicates were used. Student’s *t*-test was performed and significant differences (*p* ≤ 0.05) among samples are labeled with different letters. The error bars represent SD.

Prior to analyzing the transcript levels of different salt-responsive genes, we first quantified *VOZ1* and *VOZ2* transcripts in WT seedlings grown on medium supplemented with 0, 50, 100 and 150 mM NaCl. Alterations in transcript levels of *VOZ1* and *VOZ2* under salt stress were observed. The expression of both *VOZ1* and *VOZ2* was significantly enhanced with increasing concentration of NaCl (Figure 8). For example, at 100 mM NaCl, ~2.5- and 2.0-fold increases in transcript levels of *VOZ1* and *VOZ2,* respectively, were observed. However, 150 mM NaCl reduced the salt-induced elevation of transcript levels of *VOZs*, probably due to severe growth inhibition at this concentration. To gain further insights into the role of VOZs in salt stress, the expression level of salt-responsive genes under both “enrichment of salt-responsive genes” and the GO category of “response to salt stimulus” was compared in WT, *DKO* and COMP2-4 RNA-Seq data. The majority of salt-responsive genes were represented in *DKO* and COMP2-4 datasets and their expression profiles were opposite to each other (Figure S5 and Table S3a,b). Motif analysis of upstream (−1000 bp) regions of these genes indicated significant enrichment (*p* < 0.0001) for VOZ binding sites, viz., G-box (*ACGTG)* and NAC bind sites (*CGT[GA])* and LS-7 (*ACGT)* (Figure S6a,b (lower panels) and Table S3). Arabidopsis genes (At1g16850 (transmembrane protein), At5g59310 (LTP4), At2g37760 (AKR4c8), At5g59820 (ZAT12), At4g23600 (COR13), At5g24770 (VSP2), At1g10585 (bHLH DNA-binding superfamily protein), At2g43510 (ATTI1) and At4g37990 (ATCAD8)), which are closest to genes involved in salt tolerance in other plant species [18,19,20,21,22,23,24,25,26,27,28] and also contain either of VOZ-binding motifs in their promoter (Table S3a,b), were selected as representatives to analyze their expression under control and salt stress conditions. The expression pattern of these nine genes was analyzed by RT-qPCR. Under control conditions, the expression levels of all nine genes were significantly higher in *DKO* as compared to WT or COMP2-4 in 30-day-old plants (Additional File 1). However, in 15-day-old seedlings there was no up-regulation of salt-responsive genes in the *DKO* in untreated seedlings, suggesting differential regulation of these genes by VOZs depending on the developmental stage of the plants. Upon exposure to salt (100 mM), expression of these genes was highly induced in the WT seedlings (Figure 9). However, loss of both VOZs (*DKO*) caused a significant reduction in salt induction of these genes, suggesting that VOZs are essential for the increased expression of these salt-responsive genes under salt stress. The expression levels of three genes, viz., At1g16850, At5g59310 and At3g04720, were partially restored to WT level under salt stress in the COMP2-4 line (Figure S4). Similar to our results, expression of several cold-responsive genes that are highly expressed in the DKO were not restored in the *VOZ2* complemented line [13,14].

The majority of the salt-responsive genes contain *cis*-elements in their promoter regions to which known TFs bind. These include *CACGTG*, *CACG[G/A]C, CATGTG, VCGCGB* and *MCGTGT* that bind G_box bHLH, N_box_bHLH, Nac_box_NAC, and *CAMTA* TFs, respectively. To understand the regulation of these salt-responsive DE genes by VOZs, POBO analysis was carried out for the enrichment of these *cis-*elements in the upstream regions of salt-responsive genes. A significant enrichment (*p* < 0.0001) for *RSRE*s (*VCGCGB* and *MCGTGT*) was observed in the upstream region (−1000 bp) of the DE genes (Figure S7). Further, enrichment for *CACGTG*, but not *CACGGC* and *CATGTG,* was found in the promoter regions of the salt stress-responsive genes (Figure S6a,b—top and middle panels). Significantly, enrichment of VOZ binding consensus motifs (*CGT[GA]*, *ACGTG* and *ACGT)* was also observed (Figure S6a,b—bottom panels). Only two genes (viz. At2g37760 and At5g59820) have a canonical binding site (*ACGT_GATTCAC_ACGC*) for VOZs in their promoter regions. These results suggest that the regulation of salt-responsive genes by VOZs is accomplished via certain *cis*-elements *(CGT[GA]*, *ACGTG* and *ACGT)* within the consensus motifs of VOZs.

## 3. Discussion

Previous studies with *voz* double mutant reported several developmental defects, such as smaller plants, impaired root growth, delayed flowering, round lamina of juvenile leaves, reduced trichome number on abaxial side, and siliques with aborted seeds [11,14]. Expression of either *VOZ1* or *VOZ2* under their native promoter or expression of *VOZ2* under *CaMV35S* promoter in *DKO* completely rescued the phenotype [11,13]. In this study, we observed another phenotype. Thirty-day-old seedlings of *DKO* plants grown under day neutral conditions at 21 °C exhibited vein-clearing phenotype in older leaves (Figure 1a). This vein-clearing phenotype became more apparent with the age of the plants. Single mutants of *VOZ*s did not exhibit this developmental phenotype. Over-expression of *VOZ2* in *DKO* rescued the phenotype (Figure 1A), suggesting that VOZ1 and VOZ2 are functionally redundant in vein-clearing phenotype.

### 3.1. VOZs are Involved in Regulation of Many Stress-Responsive Genes

Our global transcriptome analysis using RNA-Seq revealed that a significant number of genes that are involved in diverse stress responses are regulated either directly or indirectly by VOZs (Figure 2a, Figure 6, Additional File 1, sheet 1,2). Previously Nakai et al. [14] and more recently Kumar et al. [12] compared the expression of genes in WT and *DKO* using microarrays. Our study significantly differs from these in several ways. Here, we used next-generation sequencing that significantly increases the depth of transcriptome analysis and avoids some problems associated with microarrays. More importantly, the use of a complemented line in which double mutant phenotypes are rescued allowed us to identify the genes that are regulated specifically by VOZs (Figure 1a,b and Additional File 1 sheet 2). Despite using RNAseq, our study revealed a smaller number of DE genes as compared to the previous studies [12,14]. This difference in the number of DE genes and limited overlap between DE genes among different studies could be due to the difference in the age and developmental stage of plants used (30-day-plants in this study vs. 14-day-old-seedlings in previous studies) and/or due to the differences in methodologies (microarrays vs RNA-Seq). In fact, developmental regulation of expression levels of TFs has been previously reported [6,29,30]. Nevertheless, our study identified a new set of 94 genes that are regulated by VOZs (Additional File 1) when compared with Nakai et al. [14] and Kumar et al. [12]. Interestingly, GO enrichment of biological process using DE genes in *DKO* from Nakai et al. [14], we found enrichment of only two biological processes, viz., farmesyl diphosphate metabolic process and sequiterpenoid biosynthetic process. This is in contrast to this study wherein we observed enrichment for 38 GO terms that are consistent with reported functions of VOZs (Figure 6a, Additional File 3). In addition, GO enrichment analysis suggested a new role for VOZs in salt stress, which was confirmed experimentally. Reproducibility among replicates, full or partial restoration of expression of ~98% of DE genes in COMP2-4 to WT level (Figure 2b) and RT-qPCR validation of expression of a number (22 genes) of randomly selected DE genes (Figure 3, Figure S5) indicates that the identified DE genes in this study are bona fide direct or indirect targets of VOZs. Enrichment of DE genes in multiple abiotic stress-responses indicates that VOZs play a major role in crosstalk between multiple stress signal transduction pathways (Figure 6b and Additional File 4). GO analysis of the DE genes indicated high enrichment of GO terms associated with diverse processes that are critical for plant responses to biotic stresses, such as bacteria and fungi, and abiotic stresses including drought, cold, salt and oxidative stress (Figure 6a). Enrichment of genes involved in response to hormones such as abscisic acid (ABA) and jasmonic acid (JA) was also observed (Figure S8 and Additional File 5). Together, these results suggest that VOZs could be integrators of a variety of stress responses. Consistent with these results, VOZs were reported to play an important role in multiple stress responses [13,14].

### 3.2. Genes with Binding Motifs for VOZs are Both Up- and Down-Regulated

Electrophoretic mobility shift assays showed that a VOZ protein binds to *GCGTN_x7_ACGC* sequence in vitro in the V-PPase gene (*AVP1*) promoter [8]. The palindromic sequence-binding site was considered as canonical VOZ TF binding site. We analyzed it to see if the promoter region (−2000 bp upstream to TSS) of DE genes is enriched for this motif, but found no significant enrichment. Only one (DE gene AT5G16360) has two VOZ binding sites (−1590, −1604 bp) in its upstream region. We followed our analysis by screening the promoter regions of DE genes with two suboptimal (*GCGTN_x7_ACG**T*** and *GCGTN_x**8**_ACGC*) and other motifs (*GCGTN_x7_AAGC*, *GCTTN_x7_ACGC*, *ACGTN _x7_ACGC*) that are reported to bind VOZs [8,12]. This analysis resulted in the identification of another DE gene (AT2G14247) containing *GCGTN_x7_ACG**T*** motif (−336 bp) and none containing *GCGTN_x8_ACGC* in their promoter regions. Recently, Kumar et al. [12] using systematic evolution of ligands by exponential enrichment (SELEX) assay and electrophoretic mobility shift assay (EMSA) not only confirmed *GCGT_GTGATAC_ACGC* as VOZ2 binding site but also revealed additional binding sites. Based on this study, we scanned the upstream region of all the DE genes and detected six additional genes (At1g23150, At2g17840, At2g22860, At2g37760, At4g00780, At5g59820) that have these new VOZ-binding sites. In addition to these *cis*-elements, other studies also identified binding of VOZs to alternate palindromic NAC-binding sequences (palNAC-BS) that are similar to other NAC proteins that are responsive to abiotic stress [10,14]. Analysis of DE genes showed that >90% contain *CGT[GA],* 58% contain *ACGTG* and 83% contain *ACGT* elements and these motifs are significantly enriched in their promoter regions (Figure 4, Table S2). Both up- and down-regulated genes showed enrichment for VOZ-binding sites (Figure 5). Significant enrichment of VOZ-binding motifs in DE genes indicates that VOZs likely regulate the expression of those genes directly by binding to these *cis*-elements.

### 3.3. VOZs Likely Regulate Expression of Some Genes Indirectly

In the promoter regions of some DE genes we did not find any of the VOZ-binding sites and these genes are likely regulated indirectly by other TFs. We found enrichment of four TF families (bHLH, C2H2, NAC and MYB-related) in DE genes (Figure 4) and TFs in three of these families were up-regulated (Figure S3). Many members of these TF families have multiple binding sites for VOZs in their promoter regions and exhibited expression levels similar to WT in the complemented line (Table S2; Additional File 1). For example, members of bHLH, NAC and C2H2 are up-regulated in *DKO* while they are down-regulated in *COMP2-4* line. In contrast, members of a MYB-related family were down-regulated in *DKO* and up-regulated in *COMP2-4*. It is possible that these TFs may regulate the expression of DE genes that do not contain canonical VOZ binding motif [12]. Together, these data indicate a complex network of regulation of expression of TFs by VOZs.

Recent studies identified *RSRE* element *VCGCGB* as the core element that is enriched in a majority of early-activated genes under stresses [31]. As this element is identical to the binding site of TFs signal responsive/calmodulin-binding transcription activators (SRS/CAMTAs) (*VCGCGB*), many studies showed SRs, in general, and SR1/CAMTA3, in particular, in regulation of multiple biotic and abiotic stress responses [5,6,32,33,34]. Significantly, SRs regulate genes involved in abiotic stress response, particularly through *MCGTGT* element. Our analysis of the promoter region of all the DE genes indicated that a significant enrichment of the *VCGCGB* and *MCGTGT* elements, suggesting that VOZs might regulate the abiotic stress responses through SRs (Figure S7). Further, the fact that the majority of these genes are misregulated in *DKO* and are implicated in various stress signaling pathways, also suggests an important role for SRs in VOZ-mediated regulation. In support of this, it has been shown that VOZs and CAMTAs interact with the *AVP1* promoter and regulate its expression [8,35]. POBO analysis indicated the enrichment of *RSRE* motif in the promoter regions of the up-regulated DE genes (Figure S7). As shown in Figure 6, significant enrichment of GO term for “responses to salt stress” and “water deprivation” was observed only in the up-regulated DE genes (Additional File 3). Further, enrichment for *VCGCGB* and *MCGTGT* in DE genes and enrichment for GO terms “water deprivation” and “cellular response to osmotic” suggest that VOZs could be regulating drought response genes through utilization of *MCGTGT* and *VCGCGB* by SRs (Figure 6, Figure S7, and Additional File 3). In fact, a significant alteration in cold and drought-responsive genes expression was observed in *DKO* line even under non-stress conditions [14]. One possibility is that VOZs form heterodimers with CAMTAs/SRs or other TFs in regulating some genes. The fact that VOZs and CAMTAs bind to the *AVP1* promoter lends supports to this. However, thus far direct interaction of VOZs and CAMTAs has not been reported. Recently, it has been shown that VOZs interacts with CONSTANS, another TF, in regulating flowering [12].

### 3.4. VOZ Confers Salt Tolerance by Activating the Expression of Salt-Responsive Genes

Double mutant line (*voz1-1 voz2-1*) were more sensitive to salt stress in terms of seed germination rate, seedling growth and root growth when compared with the WT, COMP2-4 and single mutant lines. Thus, our results suggest that (a) *VOZ1* and *VOZ2* have redundant functions in salt tolerance and (b) *VOZs* act as positive regulators of plants response to salt stress. This positive regulation of salt stress by VOZs is similar to that observed under biotic stress and differs from that of the cold and drought stress response, where it functions as a negative regulator [14]. Previously, Nakai et al. [13,14] have shown that *DKO* was significantly tolerant to cold and drought whereas it is sensitive to bacterial and fungal pathogens. They further reported that the over-expression of *VOZ2* confers tolerance to freezing and drought but curtails tolerance to biotic stresses. Taken together these results suggest that VOZs have opposing functions under salt stress as compared to cold and drought stresses. To further understand the regulation (direct versus indirect) by VOZs, salt-responsive genes were identified and subjected to POBO analysis for enrichment of *RSRE* (*VCGCGB), NAC (CGT[GA]), G-box (ACGTG)* and *ACGT* in their upstream region. This analysis revealed significant enrichment for *RSRE* (*VCGCGB*), *CGT[GA])*, *ACGTG*, *ACGT* and *MCGTGT* (Figure 5 and Figure S7). Hence, it is possible that some of these genes could be direct targets of VOZs i.e., they bind to these motifs to regulate expression. Alternatively, other TFs such as CAMTAs/SRs could also participate along with VOZs in this regulation as discussed above.

**Figure 10:**
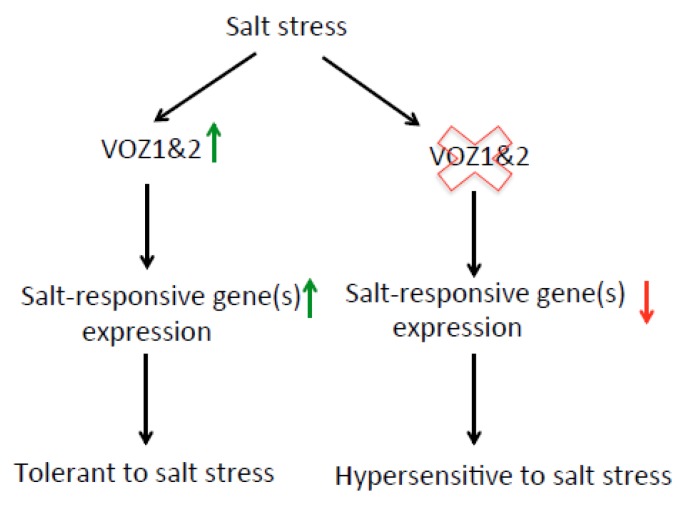
A proposed model for the role of VOZs in salt stress response (see text for details). Green and red arrows indicate the increased and decreased expression levels, respectively.

In summary, our results showed that a large number of genes associated with biotic and abiotic stress responses are regulated by VOZs. Most of these genes are likely direct targets as they contain one or more type of VOZ-binding sites in their promoter region. Analysis of DE genes suggested a new role for VOZs in salt stress. We experimentally showed that VOZs function as positive regulators of salt tolerance. The model in Figure 10 summarizes the role of VOZs in salt stress response. Plants in response to salt stress activate expression of VOZs. This activation of VOZs, in turn, regulates the expression level of salt-responsive genes either directly or indirectly thereby conferring salt tolerant phenotype. The absence of VOZs in *DKO* significantly curtails salt-induced activation of the salt-responsive genes leading to hypersensitive phenotype.

## 4. Materials and Methods

### 4.1. Plant Materials and Growth Conditions

All experiments were performed with *Arabidopsis thaliana* Columbia-0 ecotype. Seeds of single (*voz1-1*, *voz2-1*) and double mutants (*DKO*; *voz1-1 voz2-2*) of *VOZ1* and *VOZ2* used in this study were characterized previously [11]. The complemented line (COMP-4) was generated by transforming *DKO* with *VOZ2* cDNA under *CaMV35S* promoter. Plants were grown in soil in a growth chamber at 21 °C, 60% relative humidity under 12/12 h light/dark conditions.

### 4.2. Salt Stress Treatment

To study the effect of salt stress on seed germination and seedling growth, surface sterilized seeds of wild type (WT) and mutants were sown on half-strength MS medium (containing 0.5 mg/L MES and 1% sucrose) pH 5.7 and supplemented with 0, 50, 100 or 150 mM NaCl. The surface-sterilized seeds of all the lines were stratified at 4 °C in dark for 5 d prior to sowing on plates. The plates with seeds were allowed to germinate under long day condition (16 h light/8 h dark) at 22 °C. Germination rate and fresh weight of seedling and root growth was determined by recording the number of seeds that exhibited emergence of radicle, weight of the seedlings and length of the roots after two weeks of growth, respectively. All experiments were performed three times with a minimum of three replicates.

### 4.3. RNA-Seq

Total RNA from WT, *DKO* and COMP2-4 genotypes was isolated using miRNAeasy kit (Qiagen, Germantown, MD, USA#217004). Traces of genomic DNA were removed using on column DNAse digestion. RNA-Seq was performed essentially as described previously in Prasad et al. [6].

### 4.4. Mapping of the Reads and Identification of Differentially Expressed (DE) Genes

The reads were aligned to the TAIR 10 version of the Arabidopsis genome, and DE gene list was generated using the criteria as described earlier [6]. VENNY (http://bioinfogp.cnb.csic.es/tools/venny/) a web-based tool was used for identification of common genes in one or more datasets. Heatmap of DE genes was generated using log2 transformed expression values of each gene using Heatmapper [36]. Box-and-whisker plot of DE genes was generated using the log2 transformed expression values in WT, *DKO* and *COMP2*-4 with JMP Pro, version 13, statistical software (SAS, Cary, NC, USA).

### 4.5. Bioinformatics Analysis of DE Genes for VOZs Binding Motifs

Identification of DE genes containing VOZ binding motifs *GCGTN_x7_ACGC*, *ACGTG*, *CGT[GA]* and *ACGT* in their promoter was carried out using “Patmatch” (Version 1.1) tool (www. arabidopsis.org). With this tool, we identified motifs on both strands of upstream sequences (−500 bp) preceding the TSS in TAIR10 database. Both up- and down-regulated DE genes were included as input for scoring both type and number of VOZs binding motifs.

### 4.6. GO Term Enrichment Analysis

GO term enrichment analysis was performed using GeneCodis [37]. Single enrichment analysis with TAIR GO annotations was performed using the hypergeometric test with Benjamin-Hochberg false discovery rate (FDR) correction with a significance of *p* ≤ 0.05. The DE genes that are up- or down-regulated were analyzed separately.

### 4.7. Identification of TFs, Abiotic Stress and Hormone-Responsive Genes in DE List

To identify various TFs in the DE genes, a list of all TFs was obtained from Plant TF Database (version 3.0) (http://planttfdb.cbi.pku.edu.cn) [38] and all DE genes were queried against the total TF list. TAIR 10 ID of all TF genes was used as input for identifying the DE genes encoding the TFs and classifying them based on the similarity with Total TF family list. The TFs and the genes responsive to various abiotic stress conditions were obtained from STIFB (Stress Responsive TF Database) (http://caps.ncbs.res.in/stifdb2/). Promoters of the genes that contained *cis*-element for binding of the TFs that are involved in abiotic stress response were retrieved for the analysis. DE genes were queried against the list of the genes for a specific abiotic stress. Further, on the basis of overlap of locus ID (TAIR ID) between the lists of genes, they were further categorized into different subsets. Similarly, plant hormone biosynthesis and signaling genes in DE list were identified by comparing the DE genes with that of genes list of each individual hormone available from the Arabidopsis Hormone Database 2.0 (http://ahd.cbi.pku.edu.cn).

### 4.8. Promoter Analysis for Enrichment of Cis-Elements

To identify the *cis*-elements in promoters, either 500 or 1000 bp sequence upstream of the transcription start site was extracted from TAIR using an online tool for bulk sequence retrieval. For the estimation of the enrichment for particular *cis-*elements, promoter sequences (−500 or −1000 bp) were used as input for POBO analysis [39]. The upstream sequences of −500 or −1000 bp of the genes in the data set were used as an input into the web portal and analyzed for *cis*-element/motif against *Arabidopsis thaliana* background (clean). The following parameters were used for this analysis: number of promoters in cluster is equivalent to number of input sequences; number of pseudoclusters to generate =1000. For statistical significance, a linked GraphPad application calculates a two-tailed *p*-value using generated *t*-value and degrees of freedom for determination of the statistical differences between the input sequences and the background.

### 4.9. Validation of DE Genes Using RT-qPCR Analysis

Primers for validation of DE genes using Real-time qPCR (RT-qPCR) were designed using Primer Quest web tool (http://www.idtdna.com/Primerquest/Home/Index) from IDT, Coralville, IA, USA (Additional File 6). DE genes were randomly selected and analyzed for their expression levels using RT-qPCR. cDNA from 30-day-old plants was prepared and expression of each gene in all genotypes was estimated essentially as described [6]. For each genotype, cDNA from two independent biological replicates was used. Three technical replicates were used for each sample. *ROC5 (CYCLOPHILIN)* was used as a reference gene. Fold change in expression was calculated and plotted with respect to WT. The expression level in WT for each gene is considered as 1.

### 4.10. RT-qPCR Analysis of Salt-Responsive Genes

Ten-day-old control and salt-treated seedlings of different genotypes were used for extraction of total RNA. A quantity of 1 µg of RNA was used for the preparation of cDNA using Superscript III reverse transcriptase system as described in Prasad et al. [6]. The cDNA was diluted 6 times and 1.5 µL per reaction was used as a template. Expression analysis was performed using RT-qPCR as described above. The data obtained were normalized with *ACTIN2* and fold change in the expression level was calculated relative to their respective control, i.e., 0 mM NaCl. The expression level in control was considered as 1. A minimum of 3 technical replicates and 3 biological replicates were used for each experiment.

## Supplementary Materials

Supplementary information is available online at http://www.mdpi.com/1422-0067/19/12/3731/s1.

## Abbreviation

*DKO (voz1-1 voz2-1)*, *COMP2-4* (*CaMV35S: VOZ2: OCS*); DE (Differentially expressed genes).
