# Supplementary material for: Vascular Plant One-Zinc-Finger (VOZ) Transcription Factors Are Positive Regulators of Salt Tolerance in Arabidopsis
Source: Int J Mol Sci. 2018 Nov 23;19(12):3731. doi: 10.3390/ijms19123731 (PMC6321167; doi:10.3390/ijms19123731)
Supplement: Supplementary file 1 [file ijms-19-03731-s001.zip › ijms-372205-supplmentary/Figures S1-S8 .pdf]

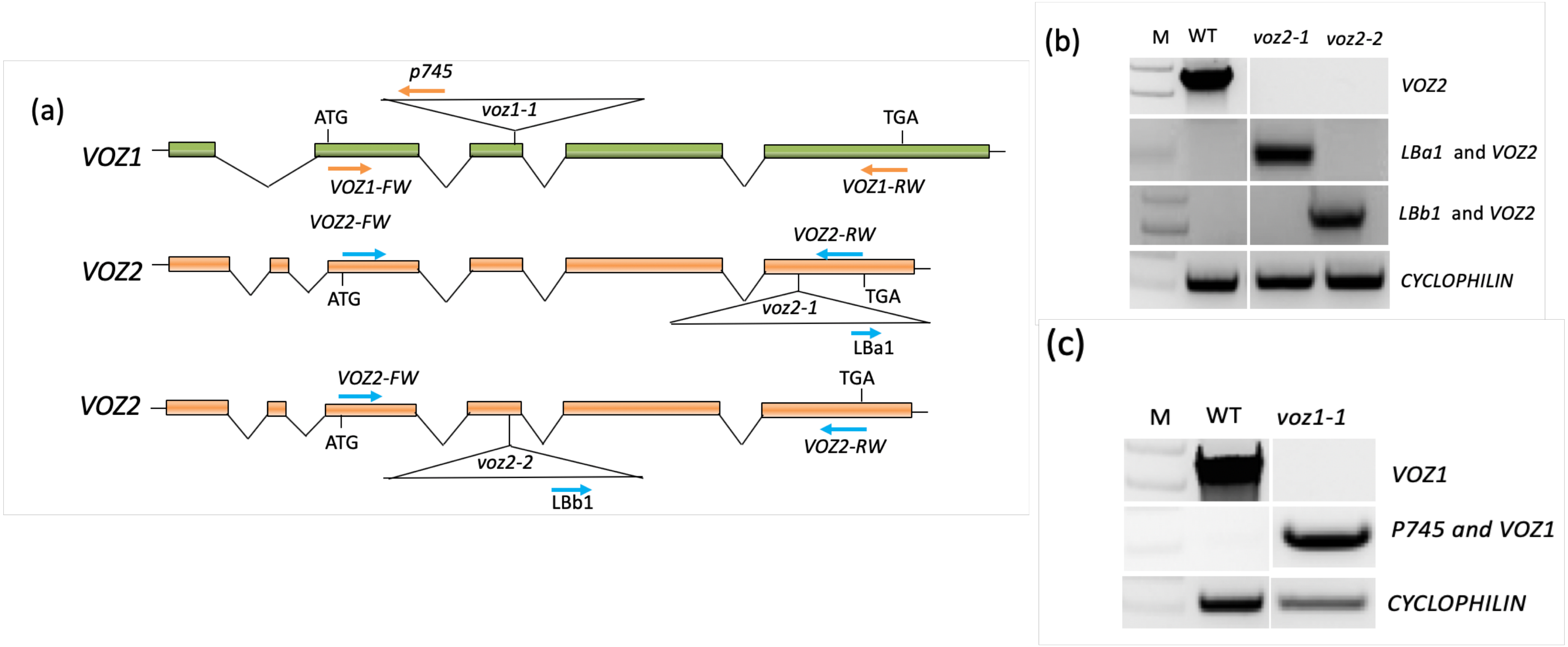

Figure S1. Genotyping of *VOZ1* and *VOZ2* single mutants**.** Prior to phenotypic analysis, genotypes were confirmed by genomic PCR. **(a)** Graphical illustrations of Arabidopsis *VOZ1* and *VOZ2* mutants. The exon are represented as boxes and introns are marked as black lines. The T-DNA insertion sites are marked by triangles. The location of each primers that were used for the genotyping are indicated by colored arrows. **(b)** Top panel (PCR with *VOZ2*-specific primers); second panel (PCR with T-DNA-specific Lba1 and *VOZ2*-specific reverse primers); third panel (PCR with T-DNA-specific LBb1 and *VOZ2*-specific reverse primers) and bottom panel (PCR with *CYCLOPHILIN*-specific primers). In all cases expected amplicon size was obtained. **(c)** Top panel (PCR with *VOZ1*-specific primers); middle panel (PCR with *Tn* insertion specific primer *P745* and *VOZ1*-specific forward primer); bottom panel (PCR with *CYCLOPHILIN*-specific primers). In all cases expected amplicon size was obtained.

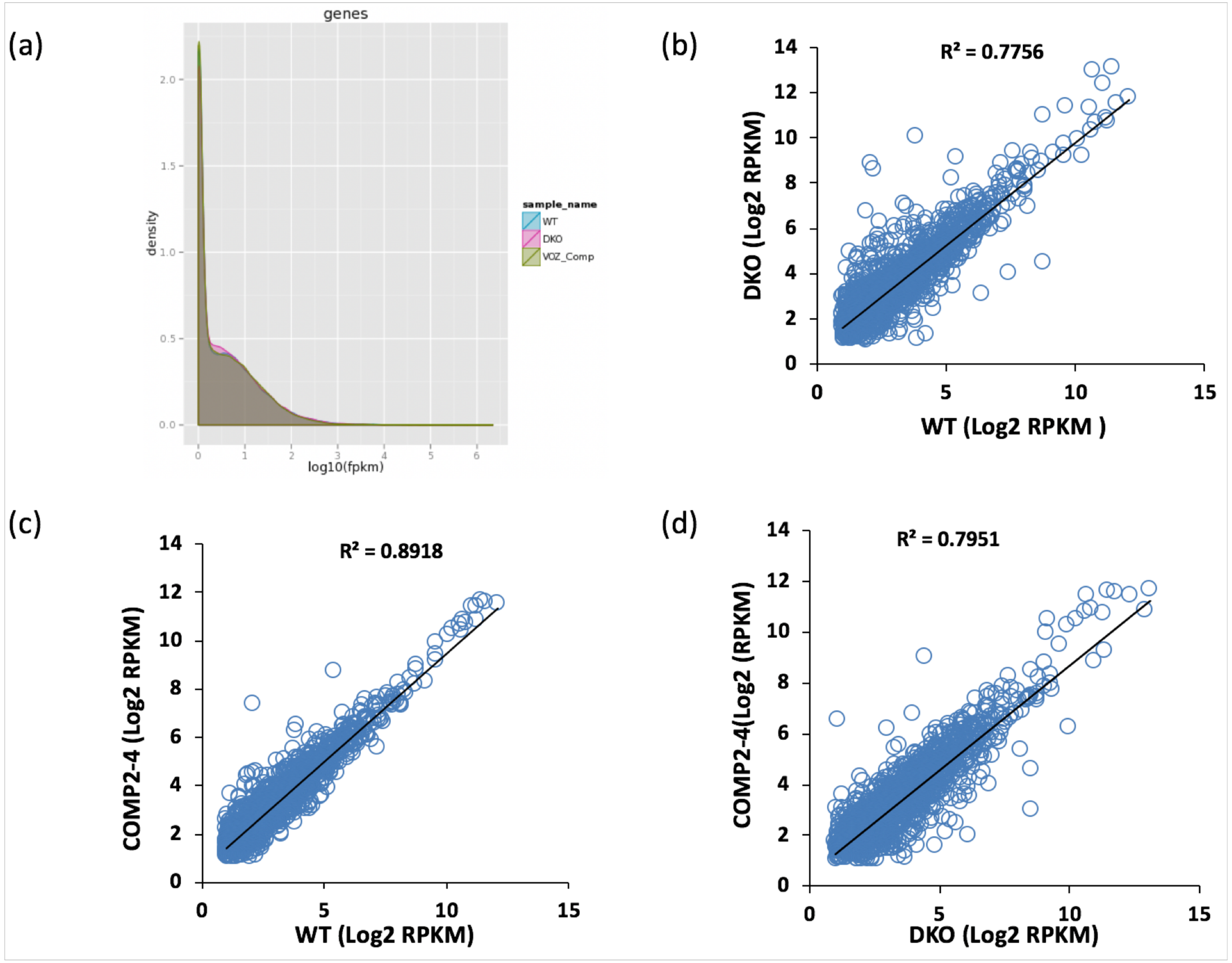

Figure S2. Scatter plots of expression values between genotypes. **(a)** Expression level distribution for all genes in WT, *DKO* and COMP2-4. **(b)** to **(d)** The average Log2 transformed values between the genotypes are plotted. In addition, the R^2 value is presented in each panel.

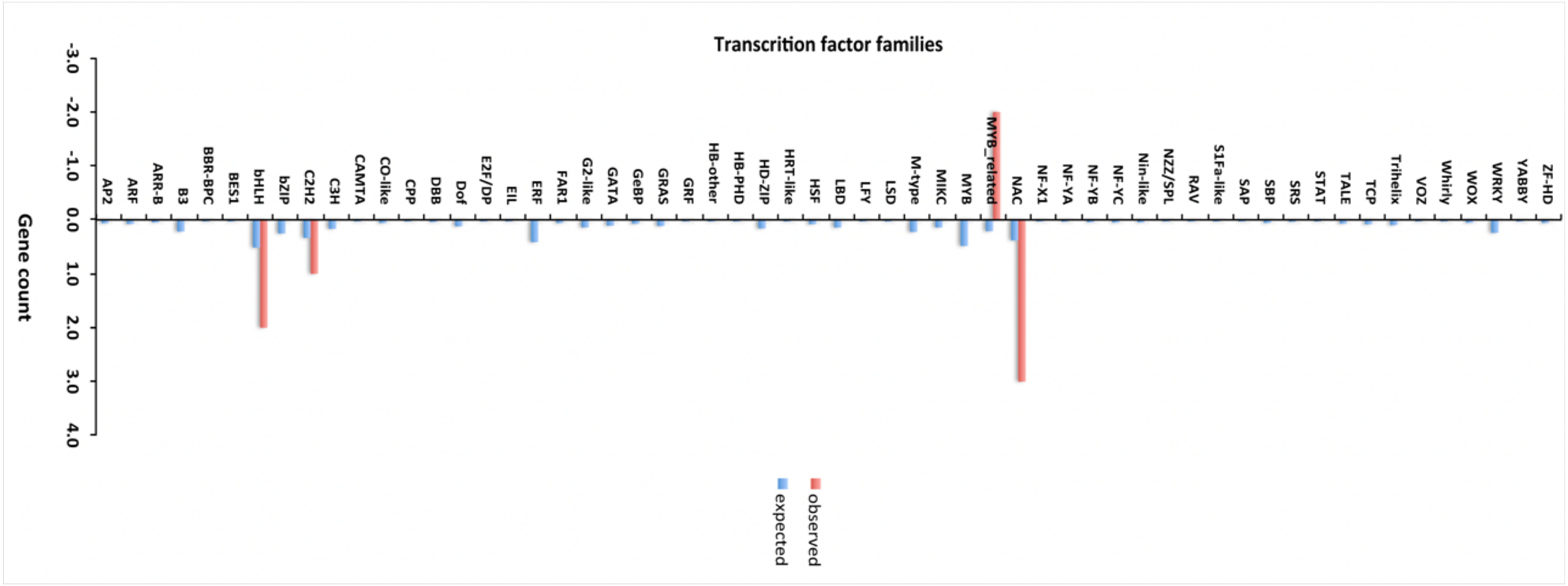

Figure S3. TF gene families in DE genes A) Enrichment of TF families in all DE genes. DE genes are enriched ($P \leq 0.05$) for specific TF families. Observed: Number of genes associated with particular TF family in DE genes. Expected: Number of genes expected in each individual TF family in the genome. Up- and down-regulated genes in each TF family are shown.

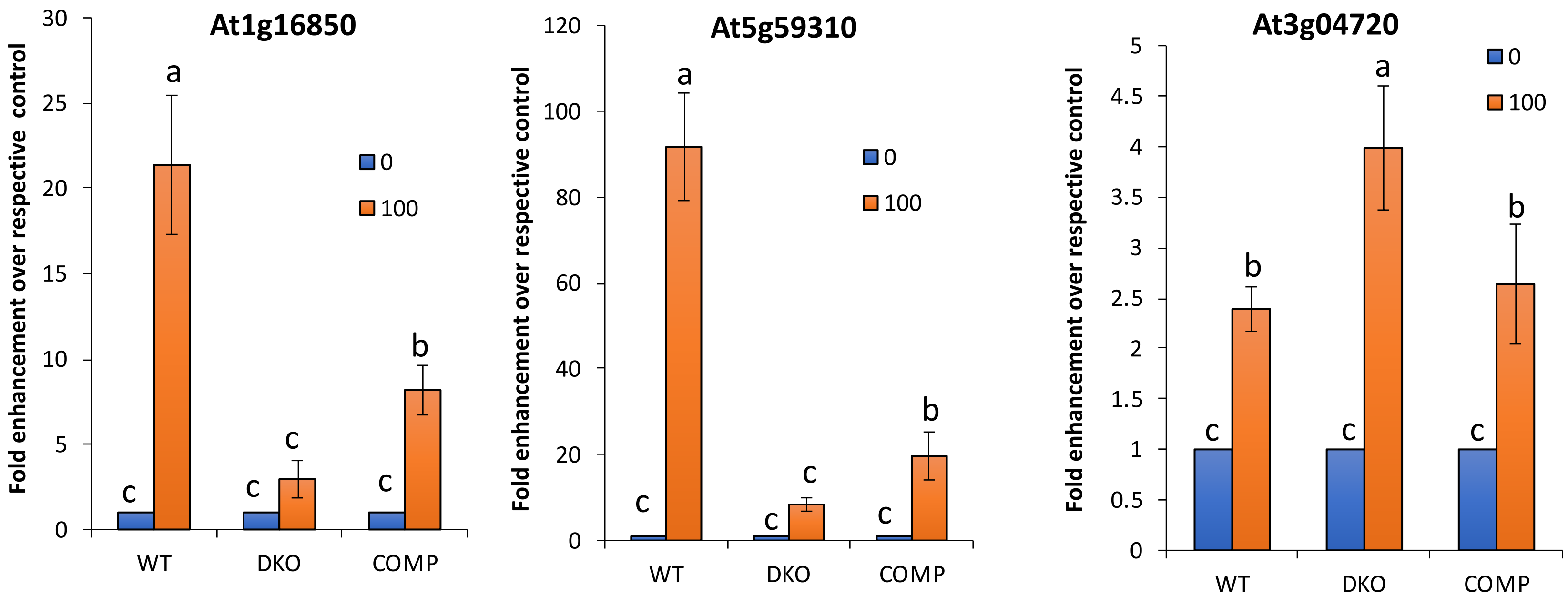

Figure S4. Restoration of the NaCl induction of salt-responsive genes in COMP2-4 line. Expression levels of salt-responsive genes in 10-day-old seedlings of COMP2-4 lines exposed to 0 and 100 mM NaCl are determined by RT-qPCR. The expression levels of salt-responsive genes were normalized with *ACTIN2*. Fold change in expression level relative to their respective controls (0 mM) is presented. 0 mM values were considered as 1. Three biological replicates were used. Student's t-test was performed and significant differences ($P \leq 0.05$) among samples are labeled with different letters. The error bars represent SD.

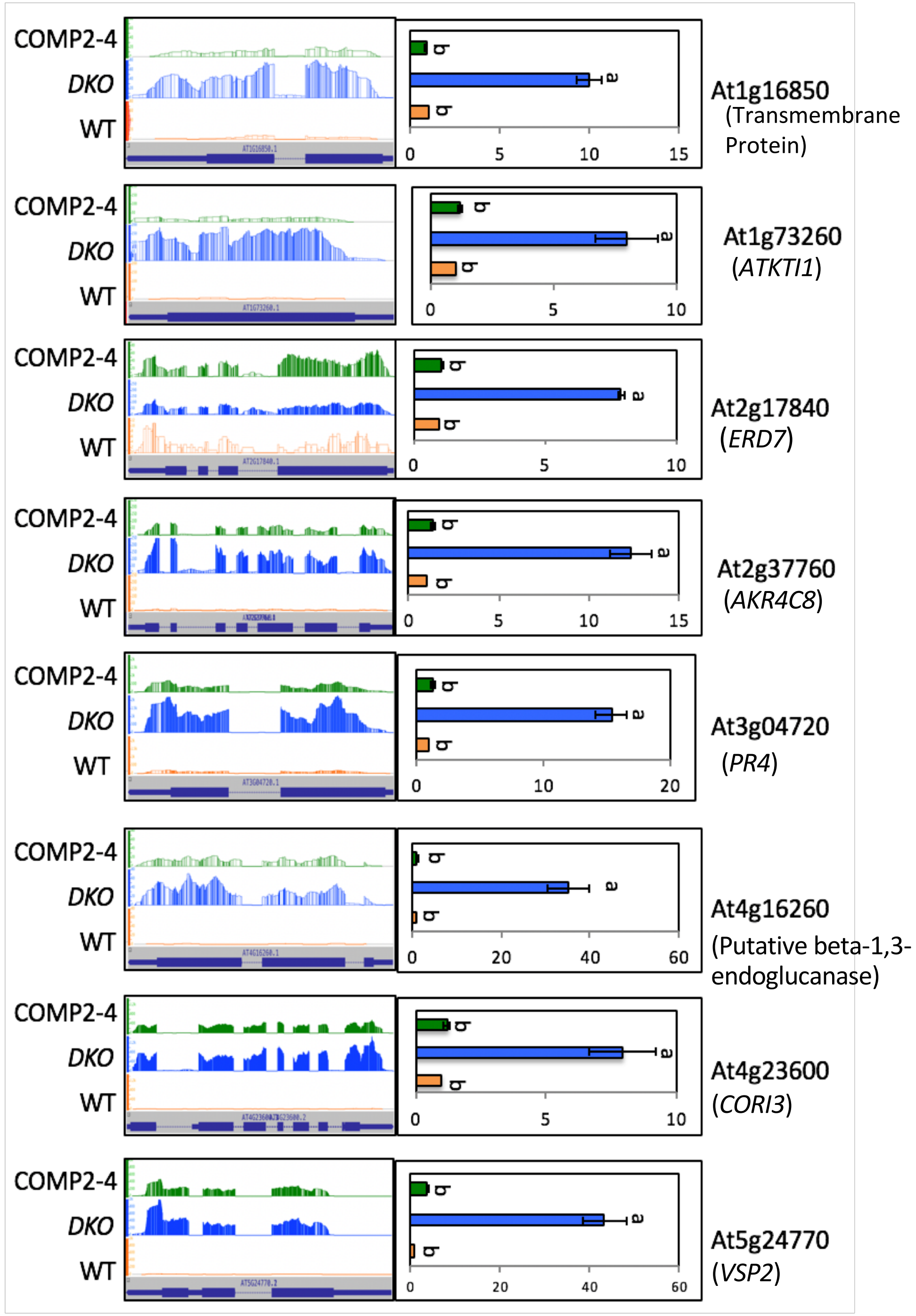

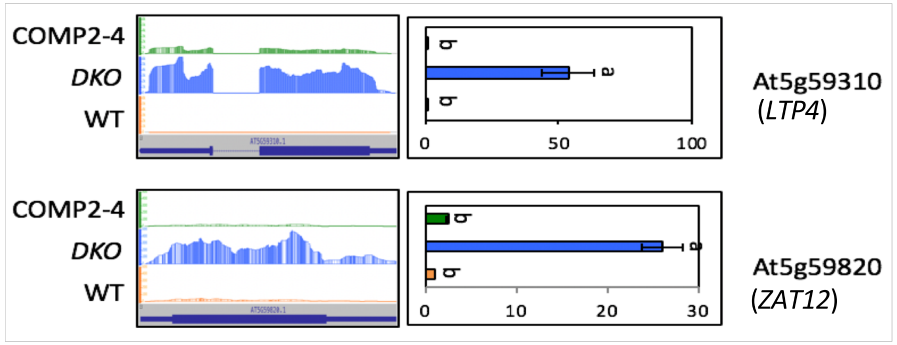

Figure S5. VOZs regulate salt-responsive genes**.** Expression levels of a few representative salt stress-responsive genes in WT, *DKO* and COMP 2-4. Left Panels: relative sequence read abundance (IGB view) as histograms in WT, *DKO* mutant (*voz1-1 voz1-2)* mutant and the complemented line (COMP2-4). The Y-axis indicates read depth with the same scale for all three lines. Right panels: Expression analysis of salt-responsive genes using RT-qPCR. Panels on right show fold change in expression level relative to WT. WT values were considered as 1. Student's t-test was performed and significant differences ($P \leq 0.05$) among samples are labeled with different letters. The error bars represent SD.

(a)

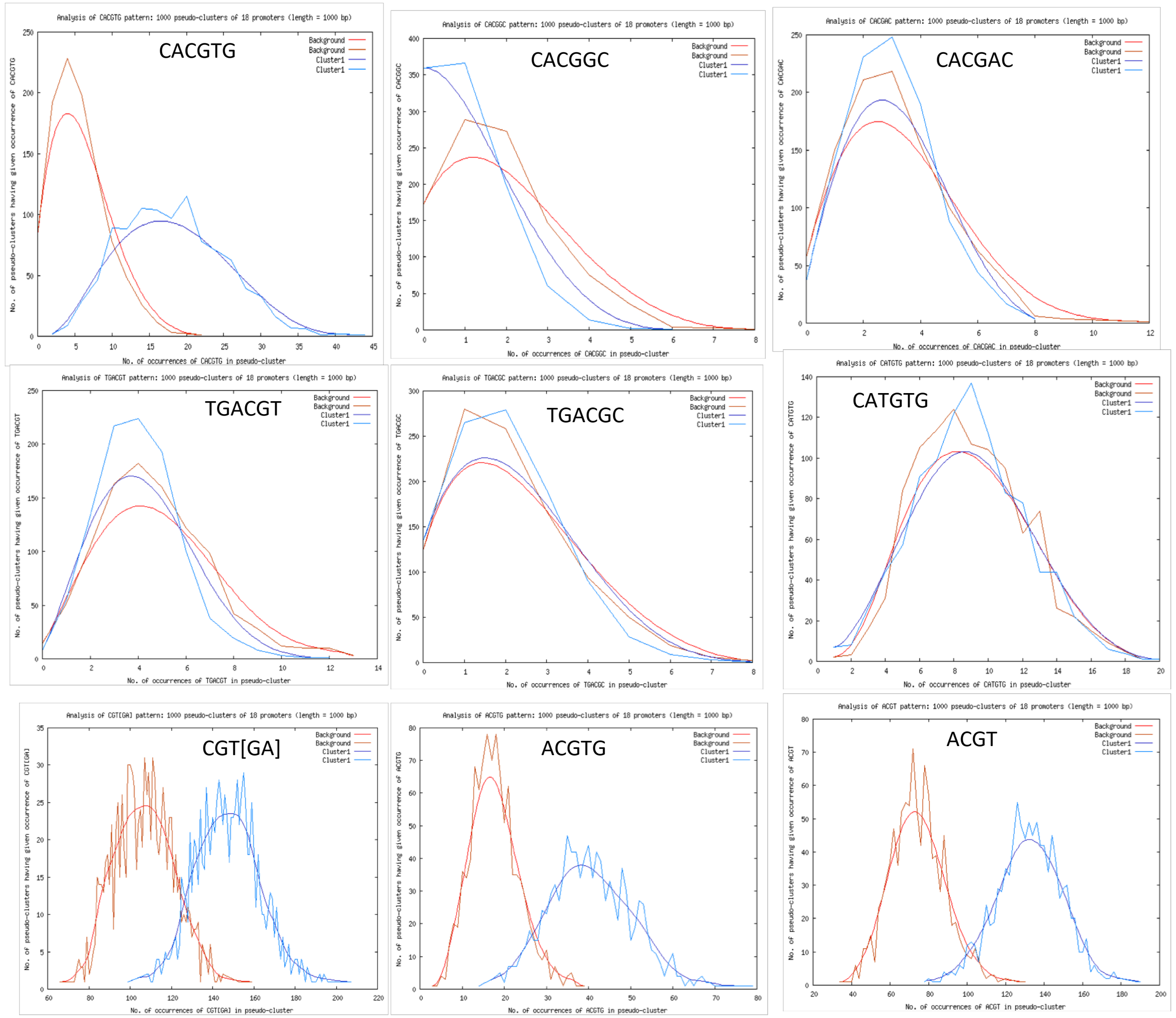

Figure S6. Promoter analysis of differentially regulated salt-responsive genes. **(a)** POBO analysis indicating the occurrence of salt-specific *cis*-elements in the upstream (-1000 bp of TSS) of salt-responsive genes (Top panel). Occurrences of VOZs recognition motifs were also plotted (Bottom panel). Data pertaining to 18 salt-responsive genes were plotted. A significant (two-tailed P $\leq$ 0.0001) enrichment of only *CACGTG (G-Box)* motif was found in the promoter region of salt-responsive genes.

(b)

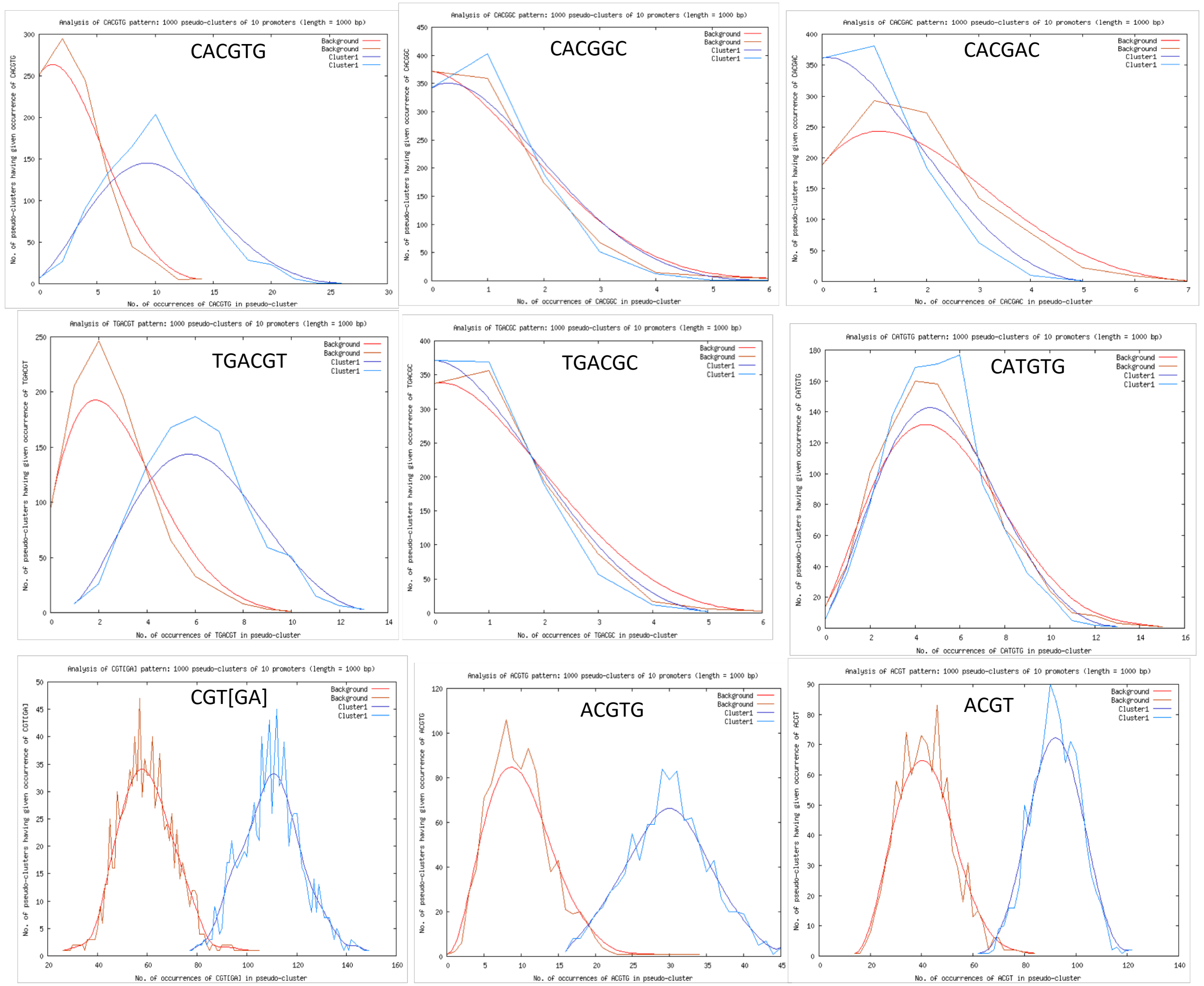

Salt, ABA and drought

ABA, drought and dehydration

VOZ binding site

Figure S6. Promoter analysis of differentially regulated salt-responsive genes. **(b)** POBO analysis indicating the occurrence of salt-specific *cis*-elements in the upstream (-1000 bp of TSS) of salt-responsive genes (Top panel). Occurrences of VOZs recognition motifs were also plotted (Bottom panel). Data pertaining to 10 salt-responsive genes were plotted. A significant (two-tailed P $\leq$ 0.0001) enrichment of only *CACGTG (G-Box)* motif was found in the promoter region of salt-responsive genes.

(a)

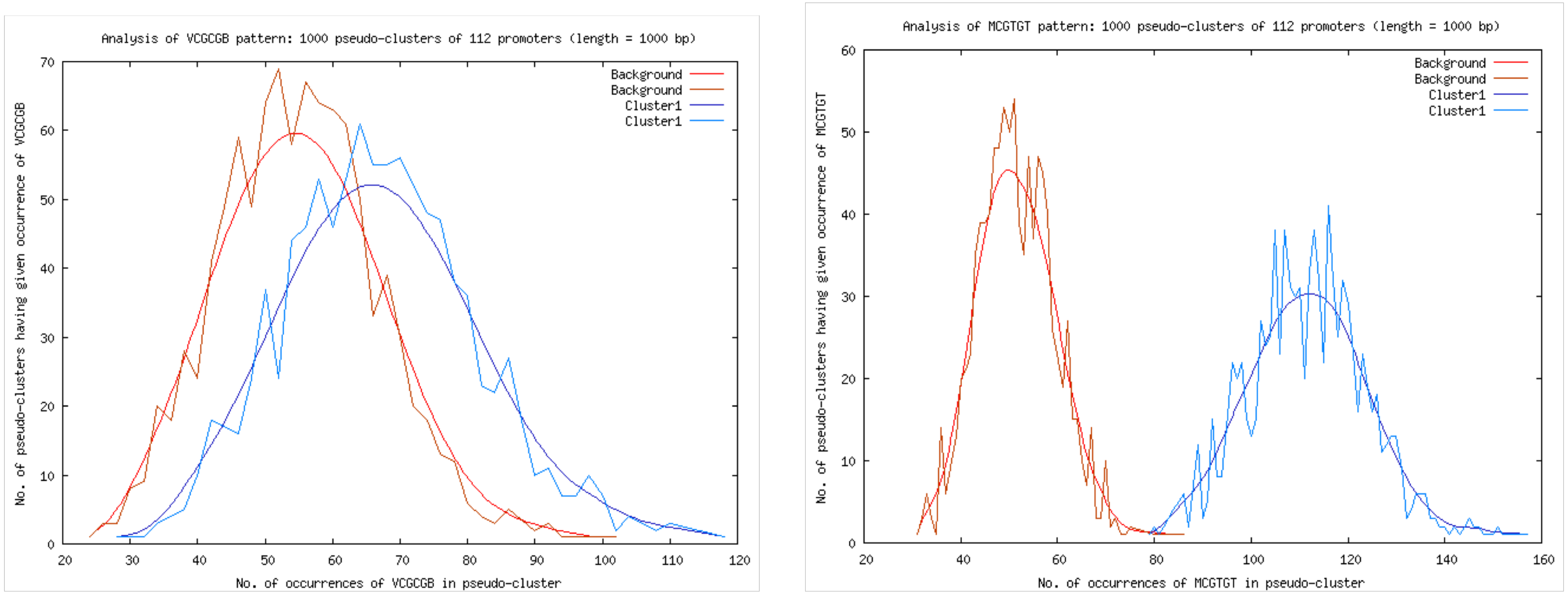

Figure S7. Analysis for RSRE element in the promoter region of DE and salt-responsive genes.
(a) POBO analysis for the occurrence of the *RSRE* (*VCGCGB*) and *MCGTGT* element in the upstream region of (-1000 bp from TSS) of all DE genes. Data pertaining to 112 DE genes were plotted. A significant (two-tailed P $\leq$ 0.0001) enrichment of *VCGCGB* and *MCGTGT* motifs was noted.

(b)

Salt regulated 18 genes (VCGCGB)

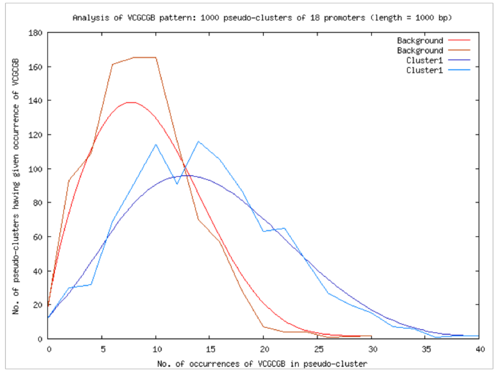

Salt regulated 18 genes (MCGTGT)

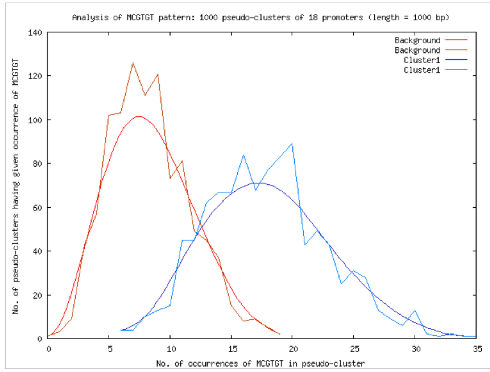

Salt regulated 10 genes (VCGCGB)

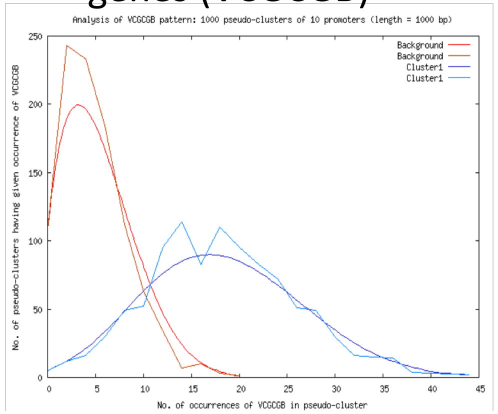

Salt regulated 10 genes (MCGTGT)

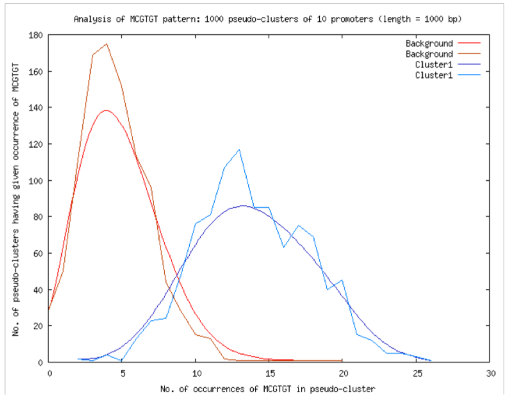

Figure S7. Analysis for RSRE element in the promoter region of DE and salt-responsive genes.
(b) POBO analysis indicating the occurrence of the *RSRE* (*VCGCGB*) and *MCGTGT* element in the upstream region of (-1000 bp from TSS) of salt-responsive genes. Data pertaining to 18 and 10 salt-responsive genes obtained in two different enrichment analyses were plotted, respectively. A significant (two-tailed $P \leq 0.0001$) enrichment of *VCGCGB* and *MCGTGT* motifs was noted.

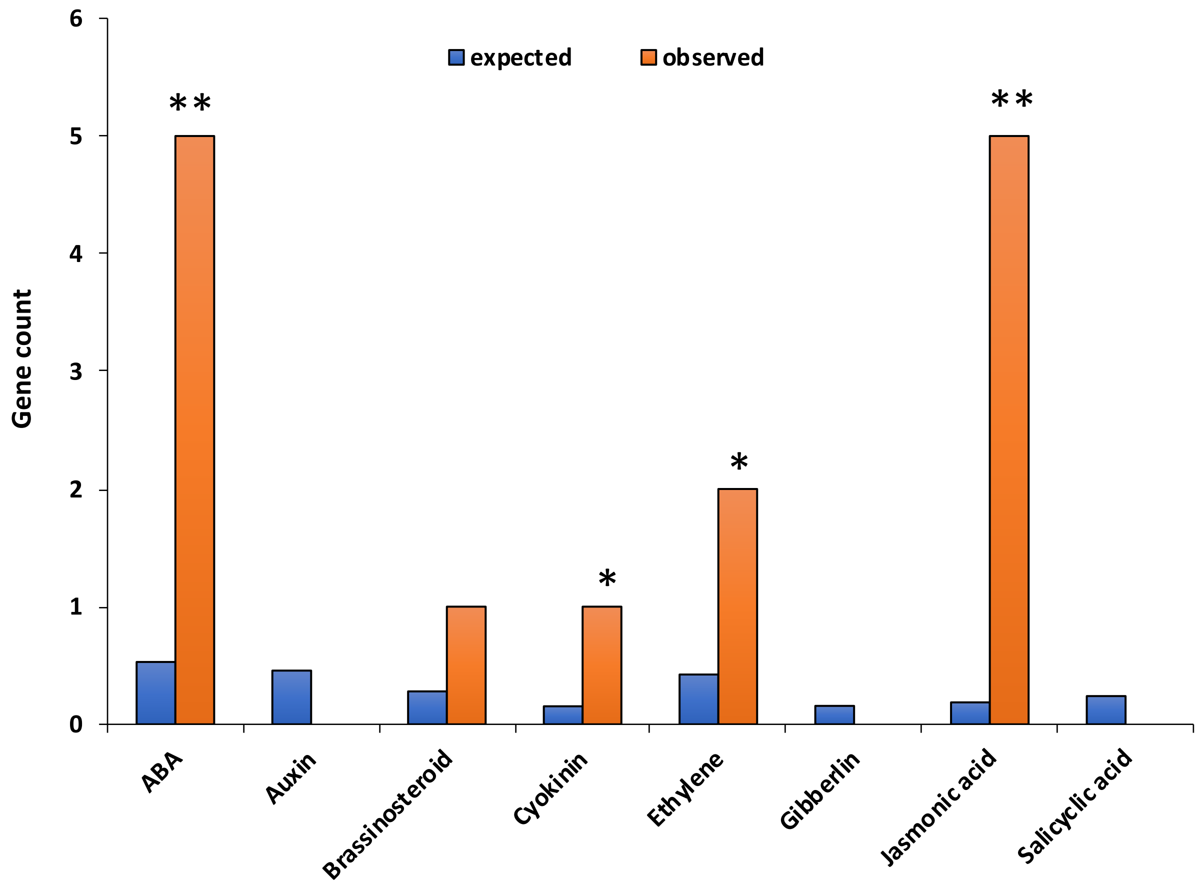

Figure S8. Enrichment of phytohormone-response pathways in DE genes. DE genes were enriched for various phytohormone-response pathways. Observed: Number of genes associated with each individual hormonal pathway. Expected: Number of genes expected to associate with each individual hormonal pathway in the whole genome. Asterisks above each bar represent significance level (** for P $\leq$ 0.0001 and * P $\leq$ 0.05).
