# Supplementary material for: Vascular Plant One-Zinc-Finger (VOZ) Transcription Factors Are Positive Regulators of Salt Tolerance in Arabidopsis
Source: Int J Mol Sci. 2018 Nov 23;19(12):3731. doi: 10.3390/ijms19123731 (PMC6321167; doi:10.3390/ijms19123731)
Supplement: Supplementary file 1 [file ijms-19-03731-s001.zip › ijms-372205-supplmentary/Tables S1-S3.docx]

**Table S1.** Summary of Illumina transcriptome sequencing from 30-d-old leaves of *Arabidopsis thaliana* plants. All reads were mapped to the Arabidopsis reference genome (TAIR 10 version). Total number of RNA–Seq reads from each sample and their mapping statistics are shown below.

| **Sample** | **Total Reads** | **Reads mapped** | **Precent Mapped** | **Uniquely Mapped** | **Percent Uniquely Mapped** | **Mutiple Hits** |
| --- | --- | --- | --- | --- | --- | --- |
| WTVOZ_R1 | 39972118 | 37634802 | 94.2 | 34068915 | 90.5 | 3565887 |
| WTVOZ_R2 | 37729617 | 35609366 | 94.4 | 32274896 | 90.6 | 3334470 |
| DKOVOZ_R1 | 75238276 | 70326172 | 93.5 | 63253484 | 89.9 | 7072688 |
| DKOVOZ_R2 | 67085386 | 63456835 | 94.6 | 56841219 | 89.6 | 6615616 |
| VOZ COMP_R1 | 39923082 | 37493531 | 93.9 | 34093479 | 90.9 | 3400052 |
| VOZCOMP_R2 | 41985763 | 39553773 | 94.2 | 35834801 | 90.6 | 3718972 |

**Table S2.** List of DE genes with different VOZ binding sites in -500bp upstream of TSS.

| S.No. | Gene ID | CGT(GA)  sites | Gene ID | ACGTG  sites | Gene ID | ACGT  sites |
| --- | --- | --- | --- | --- | --- | --- |
| \| 1 \| \| --- \| \| 2 \| \| 3 \| \| 4 \| \| 5 \| \| 6 \| \| 7 \| \| 8 \| \| 9 \| \| 10 \| \| 11 \| \| 12 \| \| 13 \| \| 14 \| \| 15 \| \| 16 \| \| 17 \| \| 18 \| \| 19 \| \| 20 \| \| 21 \| \| 22 \| \| 23 \| \| 24 \| \| 25 \| \| 26 \| \| 27 \| \| 28 \| \| 29 \| \| 30 \| \| 31 \| \| 32 \| \| 33 \| \| 34 \| \| 35 \| \| 36 \| \| 37 \| \| 38 \| \| 39 \| \| 40 \| \| 41 \| \| 42 \| \| 43 \| \| 44 \| \| 45 \| \| 46 \| \| 47 \| \| 48 \| \| 49 \| \| 50 \| \| 51 \| \| 52 \| \| 53 \| \| 54 \| \| 55 \| \| 56 \| \| 57 \| \| 58 \| \| 59 \| \| 60 \| \| 61 \| \| 62 \| \| 63 \| \| 64 \| \| 65 \| \| 66 \| \| 67 \| \| 68 \| \| 69 \| \| 70 \| \| 71 \| \| 72 \| \| 73 \| \| 74 \| \| 75 \| \| 76 \| \| 77 \| \| 78 \| \| 79 \| \| 80 \| \| 81 \| \| 82 \| \| 83 \| \| 84 \| \| 85 \| \| 86 \| \| 87 \| \| 88 \| \| 89 \| \| 90 \| \| 91 \| \| 92 \| \| 93 \| \| 94 \| \| 95 \| \| 96 \| \| 97 \| \| 98 \| \| 99 \| \| 100 \| \| 101 \| \|  \| \|  \| | \| AT2G22470 \| \| --- \| \| AT1G16850 \| \| AT1G23150 \| \| AT1G65510 \| \| AT3G27210 \| \| AT1G61800 \| \| AT1G67870 \| \| AT1G52040 \| \| AT1G73325 \| \| AT1G52890 \| \| AT2G17840 \| \| AT2G29350 \| \| AT3G02480 \| \| AT5G59820 \| \| AT1G13340 \| \| AT1G02470 \| \| AT1G10585 \| \| AT1G73260 \| \| AT1G76790 \| \| AT2G41480 \| \| AT4G11910 \| \| AT5G24780 \| \| AT1G74010 \| \| AT2G14620 \| \| AT2G37760 \| \| AT3G08860 \| \| AT1G67865 \| \| AT2G22860 \| \| AT2G39030 \| \| AT3G04000 \| \| AT4G23600 \| \| AT4G33420 \| \| AT4G39670 \| \| AT1G67860 \| \| AT1G75250 \| \| AT2G29460 \| \| AT3G44300 \| \| AT1G09500 \| \| AT1G47395 \| \| AT1G47400 \| \| AT1G65500 \| \| AT2G14247 \| \| AT2G26020 \| \| AT2G39330 \| \| AT3G03640 \| \| AT3G28210 \| \| AT3G29250 \| \| AT4G00780 \| \| AT4G18280 \| \| AT4G23680 \| \| AT4G36010 \| \| AT4G39190 \| \| AT5G13170 \| \| AT5G44420 \| \| AT5G49480 \| \| AT5G64120 \| \| AT5G66170 \| \| AT1G23130 \| \| AT1G30700 \| \| AT1G54575 \| \| AT1G64360 \| \| AT1G75270 \| \| AT2G26010 \| \| AT3G56980 \| \| AT5G02260 \| \| AT1G61120 \| \| AT2G34810 \| \| AT3G05400 \| \| AT3G44320 \| \| AT3G49580 \| \| AT4G12735 \| \| AT4G15210 \| \| AT4G16260 \| \| AT4G17470 \| \| AT5G16360 \| \| AT5G44430 \| \| AT5G59310 \| \| AT3G61210 \| \| AT4G08555 \| \| AT5G18270 \| \| AT5G23660 \| \| AT5G24770 \| \| AT1G68795 \| \| AT2G18328 \| \| AT3G09270 \| \| AT5G47060 \| \| AT3G04720 \| \| AT4G11320 \| \| AT4G16000 \| \| AT4G37990 \| \| AT5G17860 \| \| AT2G43510 \| \| AT2G43620 \| \| AT4G33550 \| \| AT5G10340 \| \| AT5G13490 \| \| AT5G22860 \| \| AT5G63130 \| \| AT1G02850 \| \| AT2G32870 \| \| AT3G26830 \| | \| 11 \| \| --- \| \| 10 \| \| 9 \| \| 9 \| \| 9 \| \| 8 \| \| 8 \| \| 7 \| \| 7 \| \| 6 \| \| 6 \| \| 6 \| \| 6 \| \| 6 \| \| 6 \| \| 5 \| \| 5 \| \| 5 \| \| 5 \| \| 5 \| \| 5 \| \| 5 \| \| 5 \| \| 5 \| \| 5 \| \| 5 \| \| 4 \| \| 4 \| \| 4 \| \| 4 \| \| 4 \| \| 4 \| \| 4 \| \| 4 \| \| 4 \| \| 4 \| \| 4 \| \| 3 \| \| 3 \| \| 3 \| \| 3 \| \| 3 \| \| 3 \| \| 3 \| \| 3 \| \| 3 \| \| 3 \| \| 3 \| \| 3 \| \| 3 \| \| 3 \| \| 3 \| \| 3 \| \| 3 \| \| 3 \| \| 3 \| \| 3 \| \| 3 \| \| 3 \| \| 3 \| \| 3 \| \| 3 \| \| 3 \| \| 3 \| \| 3 \| \| 3 \| \| 3 \| \| 2 \| \| 2 \| \| 2 \| \| 2 \| \| 2 \| \| 2 \| \| 2 \| \| 2 \| \| 2 \| \| 2 \| \| 2 \| \| 2 \| \| 2 \| \| 2 \| \| 2 \| \| 2 \| \| 2 \| \| 2 \| \| 2 \| \| 1 \| \| 1 \| \| 1 \| \| 1 \| \| 1 \| \| 1 \| \| 1 \| \| 1 \| \| 1 \| \| 1 \| \| 1 \| \| 1 \| \| 1 \| \| 1 \| \| 1 \| | \| AT3G02480 \| \| --- \| \| AT1G52040 \| \| AT2G22470 \| \| AT1G16850 \| \| AT3G27210 \| \| AT3G44320 \| \| AT1G52890 \| \| AT1G67870 \| \| AT1G73260 \| \| AT4G11910 \| \| AT4G36010 \| \| AT5G24780 \| \| AT5G66170 \| \| AT1G09500 \| \| AT1G10585 \| \| AT1G23150 \| \| AT1G65500 \| \| AT1G67865 \| \| AT1G73325 \| \| AT2G22860 \| \| AT2G39330 \| \| AT3G03640 \| \| AT3G04000 \| \| AT4G00780 \| \| AT4G12735 \| \| AT4G15210 \| \| AT4G16000 \| \| AT4G23600 \| \| AT4G39670 \| \| AT5G17860 \| \| AT5G59310 \| \| AT5G59820 \| \| AT1G02470 \| \| AT1G47395 \| \| AT1G47400 \| \| AT1G61800 \| \| AT1G65510 \| \| AT1G76790 \| \| AT2G14247 \| \| AT2G17840 \| \| AT2G26020 \| \| AT2G29350 \| \| AT2G39030 \| \| AT2G41480 \| \| AT3G04720 \| \| AT3G05400 \| \| AT3G28210 \| \| AT3G29250 \| \| AT3G49580 \| \| AT4G11320 \| \| AT4G16260 \| \| AT4G17470 \| \| AT4G18280 \| \| AT4G23680 \| \| AT4G33420 \| \| AT4G37990 \| \| AT4G39190 \| \| AT5G13170 \| \| AT5G16360 \| \| AT5G44420 \| \| AT5G44430 \| \| AT5G49480 \| \| AT5G64120 \| \| AT4G39320 \| \| AT5G40730 \| | \| 8 \| \| --- \| \| 5 \| \| 5 \| \| 4 \| \| 4 \| \| 4 \| \| 3 \| \| 3 \| \| 3 \| \| 3 \| \| 3 \| \| 3 \| \| 3 \| \| 2 \| \| 2 \| \| 2 \| \| 2 \| \| 2 \| \| 2 \| \| 2 \| \| 2 \| \| 2 \| \| 2 \| \| 2 \| \| 2 \| \| 2 \| \| 2 \| \| 2 \| \| 2 \| \| 2 \| \| 2 \| \| 2 \| \| 1 \| \| 1 \| \| 1 \| \| 1 \| \| 1 \| \| 1 \| \| 1 \| \| 1 \| \| 1 \| \| 1 \| \| 1 \| \| 1 \| \| 1 \| \| 1 \| \| 1 \| \| 1 \| \| 1 \| \| 1 \| \| 1 \| \| 1 \| \| 1 \| \| 1 \| \| 1 \| \| 1 \| \| 1 \| \| 1 \| \| 1 \| \| 1 \| \| 1 \| \| 1 \| \| 1 \| \| 1 \| \| 1 \| | \| AT3G02480 \| \| --- \| \| AT1G16850 \| \| AT1G52040 \| \| AT1G10585 \| \| AT1G61800 \| \| AT1G65510 \| \| AT1G73260 \| \| AT3G27210 \| \| AT4G23680 \| \| AT5G59820 \| \| AT2G22470 \| \| AT2G39030 \| \| AT2G41480 \| \| AT3G03640 \| \| AT4G11910 \| \| AT4G23600 \| \| AT4G36010 \| \| AT4G37990 \| \| AT4G39670 \| \| AT5G66170 \| \| AT3G61210 \| \| AT1G09500 \| \| AT1G23150 \| \| AT1G47395 \| \| AT1G47400 \| \| AT1G52890 \| \| AT1G67865 \| \| AT1G67870 \| \| AT1G73325 \| \| AT2G17840 \| \| AT2G22860 \| \| AT2G29350 \| \| AT2G39330 \| \| AT3G04000 \| \| AT3G04720 \| \| AT3G29250 \| \| AT3G44320 \| \| AT4G00780 \| \| AT4G12735 \| \| AT4G16260 \| \| AT4G17470 \| \| AT4G18280 \| \| AT4G39190 \| \| AT5G16360 \| \| AT5G24780 \| \| AT5G44420 \| \| AT5G44430 \| \| AT5G59310 \| \| AT1G64360 \| \| AT2G14620 \| \| AT2G43620 \| \| AT3G56980 \| \| AT4G08555 \| \| AT5G23660 \| \| AT4G39320 \| \| AT5G16980 \| \| AT1G02470 \| \| AT1G65500 \| \| AT1G76790 \| \| AT2G14247 \| \| AT2G26020 \| \| AT3G05400 \| \| AT3G28210 \| \| AT3G49580 \| \| AT4G11320 \| \| AT4G15210 \| \| AT4G16000 \| \| AT4G33420 \| \| AT5G17860 \| \| AT5G49480 \| \| AT5G64120 \| \| AT1G13340 \| \| AT1G23130 \| \| AT1G30700 \| \| AT1G54575 \| \| AT1G67860 \| \| AT1G74010 \| \| AT1G75250 \| \| AT1G75270 \| \| AT2G26010 \| \| AT2G37760 \| \| AT2G43510 \| \| AT3G08860 \| \| AT4G33550 \| \| AT5G02260 \| \| AT5G10340 \| \| AT5G13490 \| \| AT5G18270 \| \| AT5G22860 \| \| AT5G24770 \| \| AT3G43270 \| \| AT5G13170 \| \| AT5G63130 \| \| AT5G40730 \| | \| 12 \| \| --- \| \| 10 \| \| 10 \| \| 8 \| \| 8 \| \| 8 \| \| 8 \| \| 8 \| \| 8 \| \| 8 \| \| 6 \| \| 6 \| \| 6 \| \| 6 \| \| 6 \| \| 6 \| \| 6 \| \| 6 \| \| 6 \| \| 6 \| \| 6 \| \| 4 \| \| 4 \| \| 4 \| \| 4 \| \| 4 \| \| 4 \| \| 4 \| \| 4 \| \| 4 \| \| 4 \| \| 4 \| \| 4 \| \| 4 \| \| 4 \| \| 4 \| \| 4 \| \| 4 \| \| 4 \| \| 4 \| \| 4 \| \| 4 \| \| 4 \| \| 4 \| \| 4 \| \| 4 \| \| 4 \| \| 4 \| \| 4 \| \| 4 \| \| 4 \| \| 4 \| \| 4 \| \| 4 \| \| 4 \| \| 4 \| \| 2 \| \| 2 \| \| 2 \| \| 2 \| \| 2 \| \| 2 \| \| 2 \| \| 2 \| \| 2 \| \| 2 \| \| 2 \| \| 2 \| \| 2 \| \| 2 \| \| 2 \| \| 2 \| \| 2 \| \| 2 \| \| 2 \| \| 2 \| \| 2 \| \| 2 \| \| 2 \| \| 2 \| \| 2 \| \| 2 \| \| 2 \| \| 2 \| \| 2 \| \| 2 \| \| 2 \| \| 2 \| \| 2 \| \| 2 \| \| 2 \| \| 1 \| \| 1 \| \| 1 \| |

The number and type of binding sites were identified by Patmatch tool available on arabidopsis.org website using the default settings. Both up- and down-regulated DE genes were used for scoring the type and number of VOZ binding motifs.

**Table S3A.** Expression level and VOZ-binding sites in the promoter (-500 bp) region of 18 salt-responsive genes obtained by comparing the DE gene list with the salt stress genes in the stress responsive TF database (http://caps.ncbs.res.in/cgi-bin/mini/databases/stifdb/stress_matrix.cgi?se=NACL(http://caps.ncbs.res.in/stifdb/browse.html#se).

| S.No. | **Gene ID** | **# CGT[GA]** | **# ACGTG** | **#ACGT** | **Expression in DKO** | **P value** | **Expression in COMP2-4** | **P value** | **Symbol** | **Brief description** |
| --- | --- | --- | --- | --- | --- | --- | --- | --- | --- | --- |
| 1 | AT1G02850 | 1 | 0 | 0 | 3.56375 | 5.00E-05 | -0.833054 | 0.17465 | BGLU11 | beta glucosidase 11 |
| 2 | AT1G09500 | 5 | 2 | 4 | 3.91397 | 5.00E-05 | -1.44907 | 0.02025 | AT1G09500 | NAD(P)-binding Rossmann-fold superfamily protein |
| 3 | AT1G10585 | 5 | 2 | 8 | 3.65006 | 5.00E-05 | -3.41861 | 5.00E-05 | AT1G10585 | basic helix-loop-helix (bHLH) DNA-binding superfamily protein |
| 4 | AT1G16850 | 10 | 4 | 10 | 2.74173 | 0.0002 | -2.01864 | 0.00225 | AT1G16850 | transmembrane protein |
| 5 | AT2G17840 | 6 | 1 | 4 | 2.55471 | 5.00E-05 | -1.22842 | 0.04685 | ERD7 | Senescence/dehydration-associated protein-like protein |
| 6 | AT2G29460 | 4 | 0 | 0 | 2.44266 | 0.00025 | -1.42806 | 0.01715 | GSTU4 | glutathione S-transferase tau 4 |
| 7 | AT2G37760 | 5 | 0 | 2 | 2.89887 | 5.00E-05 | -1.4199 | 0.0222 | AKR4C8 | NAD(P)-linked oxidoreductase superfamily protein |
| 8 | AT2G43510 | 1 | 0 | 2 | 3.23833 | 5.00E-05 | -2.67732 | 5.00E-05 | TI1 | trypsin inhibitor protein 1 |
| 9 | AT2G43620 | 1 | 0 | 4 | 4.56162 | 5.00E-05 | -2.41282 | 0.00065 | AT2G43620 | Chitinase family protein |
| 10 | AT3G02480 | 6 | 8 | 12 | 2.95628 | 0.00025 | -1.48103 | 0.0322 | AT3G02480 | Late embryogenesis abundant protein (LEA) family protein |
| 11 | AT3G04000 | 4 | 2 | 4 | 2.63846 | 0.00015 | -1.73229 | 0.01215 | AT3G04000 | NAD(P)-binding Rossmann-fold superfamily protein |
| 12 | AT3G44300 | 4 | 0 | 0 | 4.25694 | 5.00E-05 | -1.91283 | 0.0105 | NIT2 | nitrilase 2 |
| 13 | AT4G18280 | 3 | 1 | 4 | 2.54088 | 0.00025 | -2.06299 | 0.00335 | AT4G18280 | glycine-rich cell wall protein-like protein |
| 14 | AT4G23600 | 4 | 2 | 6 | 3.52639 | 5.00E-05 | -0.748581 | 0.33575 | CORI3 | Tyrosine transaminase family protein |
| 15 | AT4G37990 | 1 | 1 | 6 | 5.11591 | 5.00E-05 | -4.21711 | 5.00E-05 | ELI3-2 | cinnamyl alcohol dehydrogenase 8 |
| 16 | AT4G39670 | 4 | 2 | 6 | 2.84463 | 5.00E-05 | -1.5238 | 0.01325 | AT4G39670 | Glycolipid transfer protein (GLTP) family protein |
| 17 | AT5G22860 | 1 | 0 | 2 | 2.0846 | 0.00025 | -0.676525 | 0.2651 | AT5G22860 | Serine carboxypeptidase S28 family protein |
| 18 | AT5G24770 | 2 | 0 | 2 | 4.5009 | 5.00E-05 | -0.481175 | 0.51695 | VSP2 | vegetative storage protein 2 |

**Table S3B.** Expression level and VOZ-binding site in the promoter (-500 bp) region of 10 salt-responsive genes obtained in GO term enrichment analysis. .

| S.No. | **Gene ID** | **# CGT[GA]** |  | **# ACGTG** | **#ACGT** | **Expression in DKO** | **P value** | **Expression in COMP2-4** | **P value** | **Symbol** | **Brief description** |
| --- | --- | --- | --- | --- | --- | --- | --- | --- | --- | --- | --- |
| 1 | AT1G16850 | 10 |  | 4 | 10 | 2.74173 | 0.0002 | -2.01864 | 0.00225 | AT1G16850 | transmembrane protein |
| 2 | At1g73260 | 5 |  | 3 | 8 | 3.04043 | 5.00E-05 | -3.0642 | 5.00E-05 | KTI1 | kunitz trypsin inhibitor 1 |
| 3 | AT2G17840 | 6 |  | 1 | 4 | 2.55471 | 5.00E-05 | -1.22842 | 0.04685 | ERD7 | Senescence/dehydration-associated protein-like protein |
| 4 | AT2G37760 | 5 |  | 0 | 2 | 2.89887 | 5.00E-05 | -1.4199 | 0.0222 | AKR4C8 | NAD(P)-linked oxidoreductase superfamily protein |
| 5 | At3g04720 | 1 |  | 1 | 4 | 2.52442 | 0.0002 | -1.75782 | 0.0122 | PR4 | pathogenesis-related 4 |
| 6 | At4g16260 | 2 |  | 1 | 4 | 4.38504 | 5.00E-05 | -2.08327 | 0.00185 | AT4G16260 | Glycosyl hydrolase superfamily protein |
| 7 | AT4G23600 | 4 |  | 2 | 6 | 3.52639 | 5.00E-05 | -0.748581 | 0.33575 | CORI3 | Tyrosine transaminase family protein |
| 8 | AT5G24770 | 2 |  | 0 | 2 | 4.5009 | 5.00E-05 | -0.481175 | 0.51695 | VSP2 | vegetative storage protein 2 |
| 9 | At5g59310 | 2 |  | 2 | 4 | 6.43833 | 5.00E-05 | -1.68103 | 0.0687 | LTP4 | lipid transfer protein 4 |
| 10 | At5g59820 | 6 |  | 2 | 8 | 2.95402 | 5.00E-05 | -3.22902 | 5.00E-05 | RHL41 | C2H2-type zinc finger family protein |
